# *Mycobacterium tuberculosis* arrests host cycle at the G_1_/S transition to establish long term infection

**DOI:** 10.1371/journal.ppat.1006389

**Published:** 2017-05-22

**Authors:** Bridgette M. Cumming, Md. Aejazur Rahman, Dirk A. Lamprecht, Kyle H. Rohde, Vikram Saini, John H. Adamson, David G. Russell, Adrie J. C. Steyn

**Affiliations:** 1 Africa Health Research Institute, Durban, KwaZulu Natal, South Africa; 2 Burnett School of Biomedical Sciences, College of Medicine, University of Central Florida, Orlando, Florida, United States of America; 3 Department of Microbiology, University of Alabama at Birmingham, Birmingham, Alabama, United States of America; 4 Cornell University College of Veterinary Medicine, C5 171 Veterinary Medical Center, Ithaca, New York, United States of America; 5 Centers for AIDS Research and Free Radical Biology, University of Alabama at Birmingham, Birmingham, Alabama, United States of America; 6 School of Laboratory Medicine and Medical Sciences, Nelson R. Mandela School of Medicine, University of KwaZulu-Natal, Durban, South Africa; University of Massachusetts Medical School, UNITED STATES

## Abstract

Signals modulating the production of *Mycobacterium tuberculosis (Mtb*) virulence factors essential for establishing long-term persistent infection are unknown. The WhiB3 redox regulator is known to regulate the production of *Mtb* virulence factors, however the mechanisms of this modulation are unknown. To advance our understanding of the mechanisms involved in WhiB3 regulation, we performed *Mtb in vitro*, intraphagosomal and infected host expression analyses. Our *Mtb* expression analyses in conjunction with extracellular flux analyses demonstrated that WhiB3 maintains bioenergetic homeostasis in response to available carbon sources found *in vivo* to establish *Mtb* infection. Our infected host expression analysis indicated that WhiB3 is involved in regulation of the host cell cycle. Detailed cell-cycle analysis revealed that *Mtb* infection inhibited the macrophage G_1_/S transition, and polyketides under WhiB3 control arrested the macrophages in the G_0_-G_1_ phase. Notably, infection with the *Mtb whiB3* mutant or polyketide mutants had little effect on the macrophage cell cycle and emulated the uninfected cells. This suggests that polyketides regulated by *Mtb* WhiB3 are responsible for the cell cycle arrest observed in macrophages infected with the wild type *Mtb*. Thus, our findings demonstrate that *Mtb* WhiB3 maintains bioenergetic homeostasis to produce polyketide and lipid cyclomodulins that target the host cell cycle. This is a new mechanism whereby *Mtb* modulates the immune system by altering the host cell cycle to promote long-term persistence. This new knowledge could serve as the foundation for new host-directed therapeutic discovery efforts that target the host cell cycle.

## Author summary

*Mycobacterium tuberculosis* (*Mtb*) is responsible for the estimated 1.8 million people with tuberculosis. One of the reasons for the success of this pathogen is its ability to modulate the immune system and establish a persistent infection. The manner whereby the mycobacterium senses the environment and modulates the host immune system is poorly understood. In this study, we used transcriptional analyses of both *Mtb* and the infected macrophage to ascertain mechanisms whereby *Mtb* adapts to and resides in macrophages. We found that WhiB3, a redox sensor in *Mtb* that controls virulence lipid production, is also involved in modulating the mycobacterium’s energy metabolic pathways in response to available carbon sources. As redox homeostasis regulates the virulent lipid production in *Mtb*, and the oxido-reductive homeostasis is tightly coupled with bioenergetic homeostasis, the virulent lipid production will be dependent on bioenergetic homeostasis. From the host’s perspective, transcriptional analysis revealed that *Mtb* regulates the macrophage’s cell cycle and comprehensive cell cycle analysis indicated that *Mtb* arrested the macrophages’ cell cycle. We discovered that polyketides under *Mtb* WhiB3 control were responsible for this cell cycle arrest that will potentially modulate the immune response to this intracellular pathogen. These studies reveal a novel strategy of targeting the host cell cycle for chemotherapeutic intervention.

## Introduction

The mechanisms whereby *Mycobacterium tuberculosis* (*Mtb)* senses the host environment to maintain metabolic homeostasis to establish infection are poorly understood. Metabolic homeostasis of any cell is sustained by bioenergetic pathways, such as respiration and glycolysis, which provide the cell’s energy requirements in the form of ATP.

In the lung, which is the site of infection in pulmonary tuberculosis (TB), it was found that when the lung macrophages were depleted as a result of acute infection, the majority of repopulation occurred by stochastic cellular proliferation *in situ* in a macrophage colony-stimulating factor (CSF) and granulocyte macrophage-CSF dependent manner [1]. Interleukin-4 has also been shown to induce an increase in resident macrophage numbers beyond homeostatic levels without coincident monocyte recruitment nor increased recruitment of inflammatory cells [2]. Further studies [3, 4] suggest that macrophage proliferation contributes to normal tissue homeostasis and that macrophages can replicate at the site of inflammation. There is also evidence for *in vivo* alveolar macrophage proliferation [5, 6]. Thus, the proliferation of tissue resident lung macrophages in TB will be predisposed to modulation by *Mtb*.

*Mtb* alters essential host functions by the release of polyketides, lipids and cell wall components such as poly- and di-acyltrehaloses (PAT/DAT), phosphatidylinositol mannosides 1 & 2 (PIM 1,2) and 6 (PIM_6_), trehalose dimycolate (TDM), sulfolipids (SL-1), phenolic glycolipids (PGL-1), mycolic acids and phthiocerol dimycocerosates (PDIM) during infection [7]. *Mtb* PhoP [8] and WhiB3 [9] are key regulators of these lipids.

Other bacteria secrete or directly inject effector molecules and toxins into the host that interfere with the eukaryotic cell cycle to facilitate disease or persistence; these effector molecules have been termed cyclomodulins [10]. Cyclomodulins can be inhibitory, for example, the cytolethal distending toxin (CDT) produced by *Escherichia coli*, *Shigella dysenteriae* and *Salmonella typhi* blocks the host cell cycle at the G_2_/M transition [11] and the vacuolating cytotoxin (VacA) of *Helicobacter pylori* induces G_1_ cell cycle arrest and cell death [12]. Stimulatory cyclomodulins, including *E*. *coli* cytotoxic necrotizing factors, *Bordetella* dermonecrotic toxin, and *H*. *pylori* CagA promote cell proliferation [11]. Cyclomodulins are not always proteins as is evident by the production of the polyketide mycolactone by *Mycobacterium ulcerans* [13] and *E*. *coli* polyketides [14]. Polyketides are lipid-like molecules that are smaller than known protein toxins but have potent biological activities, for example, antibiotic (erythromycin), immunosuppressant (rapamycin) and antifungal (amphotericin B).

Previously, we have shown how *Mtb* WhiB3 affects the virulence of two pathogenic mycobacterial strains in different animal models [15]. WhiB3 is a 4Fe-4S cluster DNA binding protein that maintains intracellular redox balance by sensing host-generated NO and O_2_ [16] and modulating virulence polyketide lipids to cause disease [9]. As the cytoplasmic redox environment is tightly coupled to central metabolism, WhiB3 was implicated in regulating the mycobacterium’s metabolism. Here we hypothesized that WhiB3 maintains bioenergetic homeostasis to control production of factors that subvert host cell function. To test this hypothesis, we exploited a combination of *Mtb* and macrophage transcriptomic analyses, real-time bioenergetic flux analysis, and a series of host cell cycle analyses.

## Results

### WhiB3 regulates bioenergetic homeostasis

**Fig 1:**
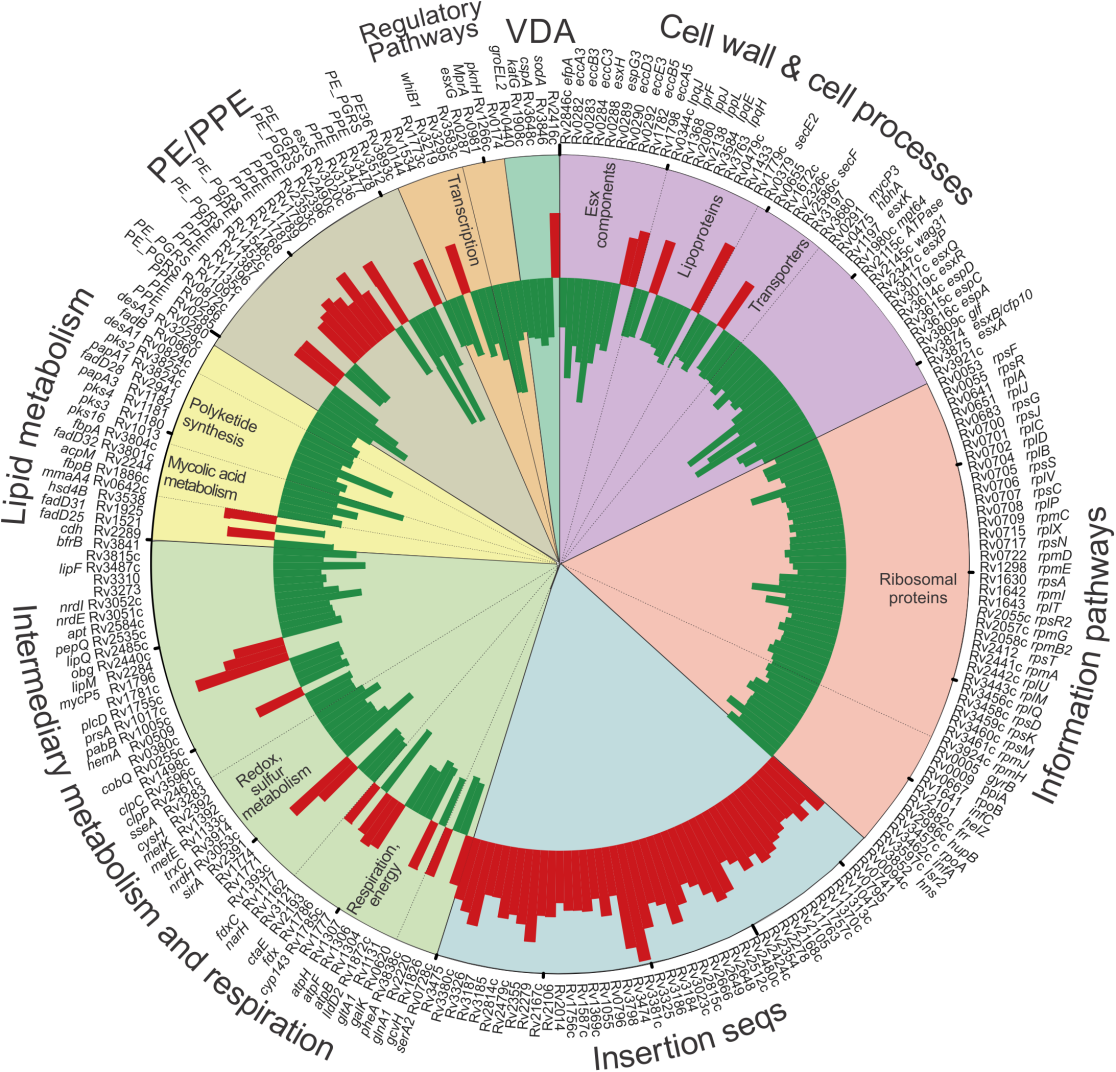
WhiB3 regulates transcription of respiration, lipid and intermediary metabolism in *Mtb*. Circos plot of fold change in gene expression in *MtbΔwhiB3* relative to that in wt *Mtb* H37Rv, p<0.05, expression ratio >1.52 or <0.66. p values were calculated with a 2-way ANOVA. Fold changes were calculated from the means of three experiments performed in triplicate. Functional group categories are depicted by the colored segments and annotated on the outer rim of the plot. Sub-categorization is annotated on the inside of the plot. Of note is that WhiB3 controls the expression of 50 genes involved in intermediary metabolism and respiration and 19 genes implicated in lipid metabolism. See S1 and S2 Tables.

To investigate mechanisms of WhiB3-mediated virulence, we examined the global transcriptome of wt *Mtb* and *MtbΔwhiB3* grown in 7H9 media to mid-log phase (Fig 1). For a complete list of the 315 WhiB3 regulated genes, see S1 Table. Notably, WhiB3 controls the expression of 50 genes involved in intermediary metabolism and respiration, of which 11 genes are concerned directly with aerobic respiration and energy metabolism (*e*.*g*., *ctaE*, *atpB*, *atpF and atpH*, *lldD2*, *gltA*). The down-regulation of ATP synthase subunits and a component of the *aa*_*33*_-type cytochrome oxidase c (CtaE) in *MtbΔwhiB3*, all essential components of oxidative phosphorylation, suggest that WhiB3 is involved in regulating bioenergetic homeostasis. Downregulation of CtaE, which functions as an H^+^ pump, would reduce the extrusion of protons across the membrane during respiration strongly suggesting a role for WhiB3 in maintaining an energized membrane, which is essential for bioenergetic homeostasis. Conversely, the gene encoding citrate synthase 3 (*gltA-1*) of the methylcitrate cycle, which generates succinate that feeds into succinate dehydrogenase, SDH, of the electron transport chain (ETC), is upregulated in *MtbΔwhiB3*. This suggests that the mutant is attempting to restore an energized membrane potential by upregulating the formation of succinate.

As previously demonstrated by our studies [9], the transcriptomic data (Fig 1) indicates that WhiB3 is involved in redox homeostasis with the downregulation of genes encoding: antioxidants, such as thioredoxin (*trxC*); electron carriers, such as ferredoxin (*fdxC*); enzymes, such as nitrate reductase (*narH*), other reductases (*hemA*, *cysH*, *nrdE*), monooxygenases (*rv1393c*) and enzymes that utilize reducing equivalents in anabolism (sulfite reductases, *sirA*, and glutamine synthase, *glnA1*).

Not surprisingly, the down-regulation of a large number ribosomal genes (Fig 1), which is essential for protein synthesis and the major consumer of cellular energy [17, 18], is consistent with a role of WhiB3 in maintaining bioenergetic homeostasis. A noticeable observation was that WhiB3 negatively regulates 43 of 56 insertion sequence (IS) loci, representing almost 30 ISs (Fig 1). Earlier studies have shown that intermediary metabolism, redox balance, and DNA metabolism controls transposition of ISs [19]. These studies have shown that the formation of transposomes is facilitated by nucleoid-associated proteins such as HNS, HU and IHF [19]. Intriguingly, WhiB3 regulates several nucleoid associated proteins including HNS, Lsr2 and HupB (Fig 1), which suggests a role for WhiB3 in the regulation of IS.

A previous transcriptomic study investigating the role of WhiB3 in acid resistance of *Mtb* [20] also found WhiB3 regulated genes involved in redox homeostasis, secretion and lipid metabolism differentially regulated in response to acidic pH; although there were only 12 significantly regulated genes in common with our transcriptomic data, primarily involved in secretion and lipid metabolism. This is expected as our microarray was performed at neutral pH, in contrast to Mehta et al. investigating differential regulation between acidic pH (4.5) and neutral pH. WhiB3 also regulates the expression of several genes belonging to the ESX-1 secretion system including, but not limited to *esxA*, *esxB*, *espA*, *espC* and *espD*, pointing to a complex role in the regulation of virulence.

In line with the WhiB3 regulation of virulence lipid anabolism previously published by Singh et al. (2009), genes encoding polyketide synthases (*pks16*, *3*, *4 and 2*), other enzymes involved with polyketide synthesis (*papA3*, *papA1*) and lipid anabolism (*mmaA4*, *fbpB*, *cdh*, *fbpA*) were downregulated in *MtbΔwhiB3*. The microarray data were validated by performing Q-PCR on select genes regulated by WhiB3 (S2 Table). In sum, the genes involved in intermediary metabolism and respiration, lipid metabolism, translation, transposition and secretion provide fresh insight into the role WhiB3 plays in virulence.

**Fig 2:**
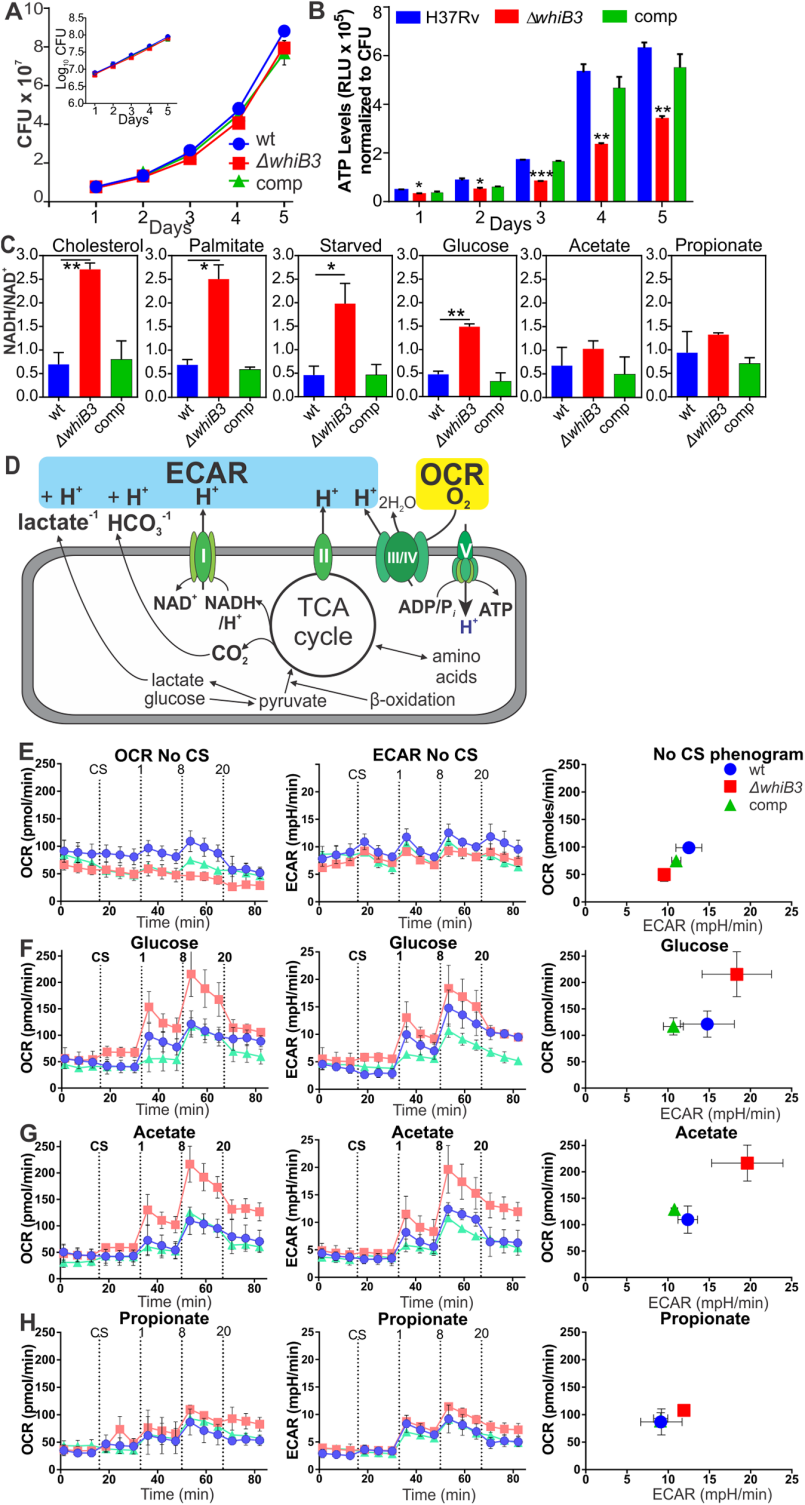
WhiB3 regulates ATP levels, NADH/NAD^+^ ratios and bioenergetic homeostasis in *Mtb*. **(A)** Growth curves/rates for wt *Mtb* H37Rv, *MtbΔwhiB3*, and the complemented strain (comp) are matching as indicated by CFU over 5 days. Inset: growth rate in logarithmic format. **(B)** ATP levels in *MtbΔwhiB3* were significantly lower than those in wt *Mtb* and the complemented strain on Day 5 as measured by luminescence (RLU) normalized to CFU. Error bars of **(A)** and **(B)** represent SEM of the mean of two independent experiments performed in duplicate. *p<0.01 (Unpaired Student’s *t* test). **(C)** NADH/NAD^+^ ratios of *Mtb* strains grown in 50 μM of the indicated carbon sources until an OD_600_ of 0.8. Error bars represent SD of the mean of at least three independent cultures, *p<0.05, **p<0.001 (Unpaired Student’s *t* test). **(D)** Schematic representation of *Mtb* extracellular flux measured by the XF96. **(E-H)** OCR, ECAR and a phenogram of wt *Mtb* (blue), *MtbΔwhiB3* (red) and the complemented strain (green) after the addition of one of the following carbon sources (CS): **(E)** no carbon sources, **(F)** 50 μM glucose, **(G)** 50 μM acetate or **(H)** 50 μM propionate before and after addition of 1, 8 and 20 μM CCCP. Error bars represent SD from the mean of triplicate wells. Data are representative of three independent experiments.

In support of WhiB3 regulation of bioenergetics (Fig 1), the ATP concentration in *MtbΔwhiB*3 was found to be significantly lower (p<0.0001) than that in wt *Mtb* and the complemented strain over a five-day growth period (Fig 2B), despite all three strains having matching growth rates (Fig 2A).

NADH feeds electrons into the electron transport chain at complex I and is essential for bioenergetic homeostasis. Using LC-MS/MS, significantly higher NADH/NAD^+^ ratios were observed in *MtbΔwhiB*3 in comparison to wt and the complemented strain when the bacilli were grown in the presence of physiological concentrations of cholesterol, palmitate, glucose, or no carbon sources for 24 h whereas no significant differences were noted when grown on acetate or propionate (Fig 2C). The increased NADH/NAD^+^ ratio in *MtbΔwhiB*3 grown in glucose correlates with the lower ATP levels in the WhiB3 mutant. This reaffirmed that WhiB3 maintains the intrabacterial NADH/NAD^+^ poise, but demonstrates it in light of the ability of WhiB3 to regulate the metabolic switch in response to available carbon sources [9]. Also, it indicates that WhiB3 indirectly regulates the donation of electrons into the ETC by controlling intracellular levels of NADH that donate electrons to menaquinone via Complex I (NADH dehydrogenase) into the ETC to sustain oxidative phosphorylation (OXPHOS).

The influence of WhiB3 on mycobacterial bioenergetics was determined by examining extracellular flux of *Mtb*, *MtbΔwhiB3* and the complemented strain using a XF96 Extracellular Flux Analyzer [21]. This analyzer allows continuous real-time quantification of O_2_ consumption and extracellular acidification of multiple samples with high sensitivity in a non-invasive manner [21]. The mycobacteria were adhered to each well in specialized microplates and fluorophore-based O_2_ and H^+^ sensors measured their oxygen consumption rate (OCR) in pmol O_2_ per minute, which gives a measure of OXPHOS, and extracellular acidification rate (ECAR) in mpH units per minute, which represents carbon catabolism, respectively (Fig 2D). Plots of OCR versus ECAR, known as phenograms, give an overall illustration of the energy phenotype of the cells. Initially, extracellular flux of the three strains was measured in 7H9 media without a carbon source (CS) and after the addition of glucose, acetate, or propionate. No significant changes were observed in the OCR or ECAR before and after the addition of the carbon source to all three strains, apart from glucose, where there was a slight increase in OCR of *MtbΔwhiB3* after the addition of glucose (Fig 2E–2H). To address the capacity of the mycobacteria to cope under bioenergetic stress when growing in different CSs, increasing concentrations of the membrane uncoupler, CCCP, were added. When no CS was present, the *whiB3* mutant had no capacity to increase OCR in response to the bioenergetic stress induced by CCCP (Fig 2E). In the presence of glucose or acetate, after the addition of 8 μM CCCP, OCR and ECAR of the *MtbΔwhiB3* increased significantly more than OCR and ECAR of the wt and complemented strains (Fig 2F and 2G). This is particularly noticeable in the phenograms of glucose and acetate. However, in the presence of propionate (Fig 2H), no differences were observed among all three strains after the addition of 8 μM CCCP. This provides evidence for a role of WhiB3 in regulating the metabolic switch and in turn energy metabolism in response to available environmental carbon sources in the presence of bioenergetics stress. In sum, the microarray, ATP concentrations, NADH/NAD^+^ ratios and the extracellular flux analysis suggest *Mtb* WhiB3 is necessary for maintaining bioenergetic equilibrium.

### Role of WhiB3 in adaptation and growth of *Mtb* in the macrophage phagosome

**Fig 3:**
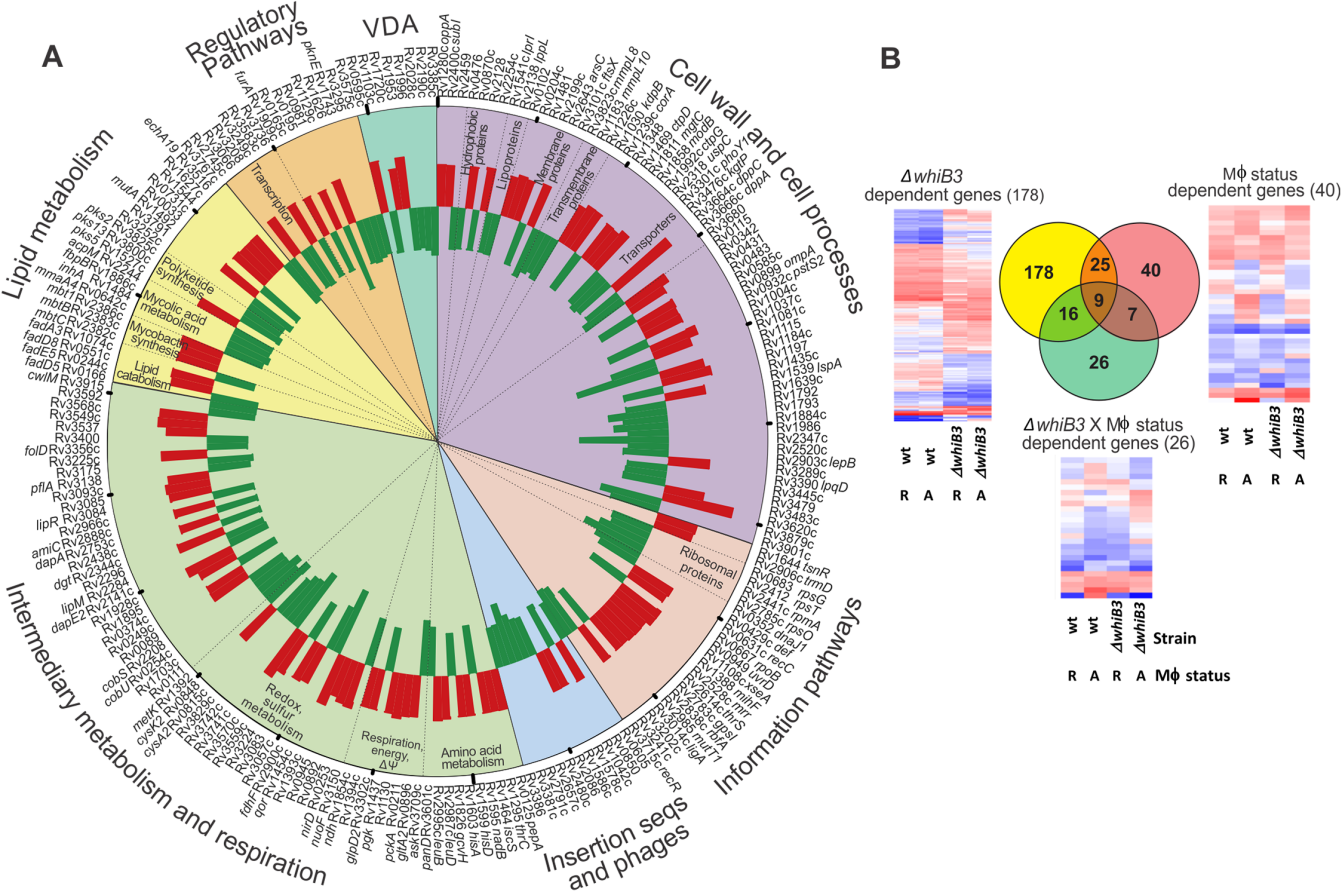
Role of WhiB3 in establishment of mycobacterial infection in macrophages. **(A)** Circos plot of WhiB3-dependent responses in *Mtb* to macrophage infection, p<0.05 (>1.2x fold change). p values were calculated with the one-way ANOVA. Fold changes were calculated from the mean of four biological replicates. The array design compared transcript levels of intramacrophage bacilli (*Mtb* and *MtbΔwhiB3*) to strain-matched extracellular controls. Functional group categories are depicted by the colored segments and annotated on the outer rim of the plot. Sub-categorization is annotated on the inside of the plot. See S3 Table. **(B)** Impact of WhiB3 and macrophage activation on *Mtb* intracellular gene expression. Venn diagram showing overlap of genes whose expression is significantly altered due to strain (Yellow: wt *Mtb* or *MtbΔwhiB3*) or macrophage status (Red: resting versus activated) or both parameters (Green). Genes with significantly altered expression in response to at least one parameter (p<0.01) were identified by 2-way ANOVA. Only genes whose array signals passed quality filters in all three conditions were included. This illustrates the dominant impact of WhiB3 on the transcriptional adaptation of *Mtb* in macrophage phagosomes. Heat maps of the expression of the genes differentially regulated by the strain alone, macrophage status alone (R, resting; A, activated) or a combination of both parameters are depicted.

**Fig 4:**
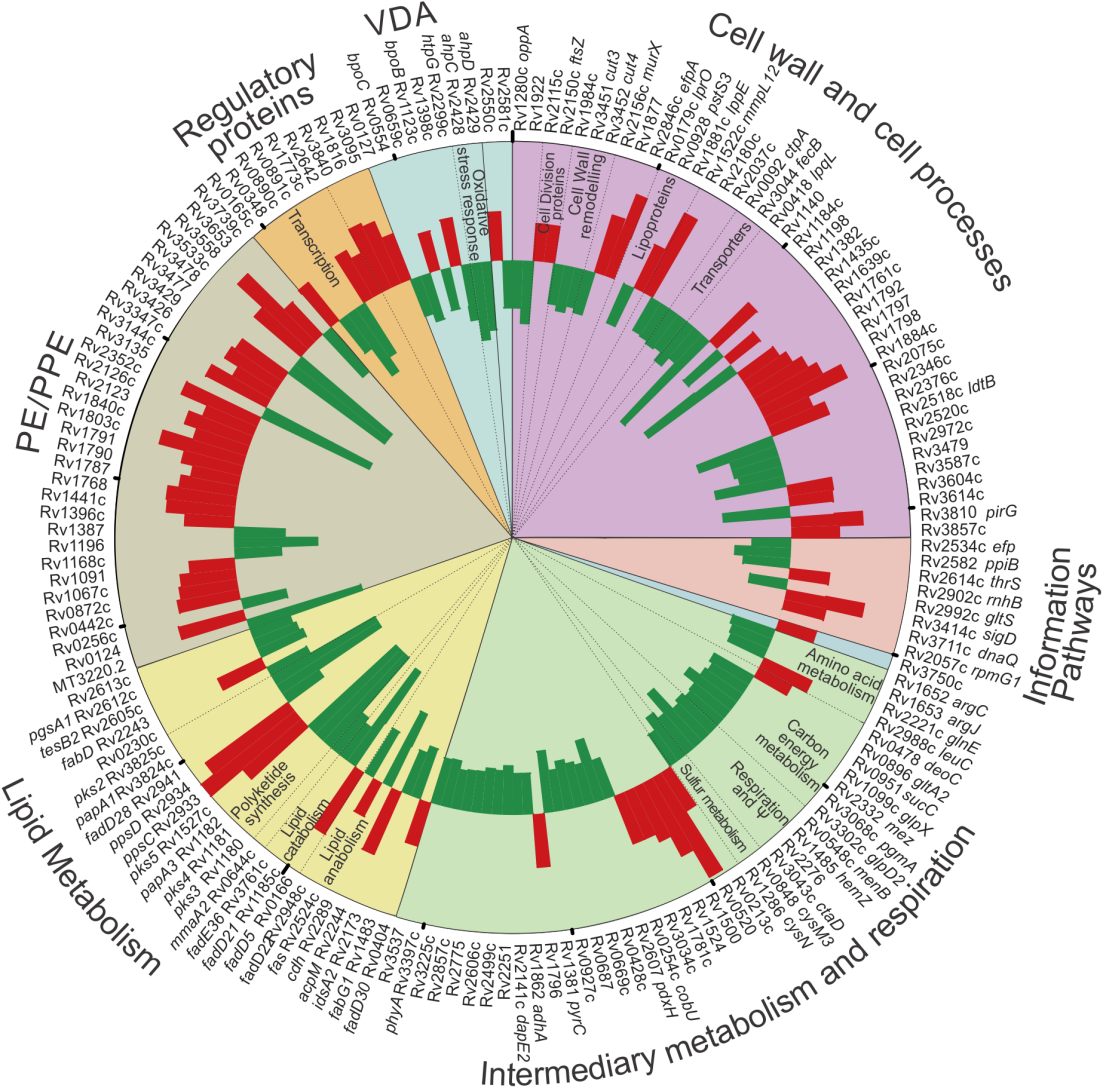
Role of WhiB3 in establishment of mycobacterial infection in macrophages. Circos plot of WhiB3-dependent responses to intraphagosomal survival, p<0.05 (One way Anova) (>1.2x fold change) in BMDM. The analysis made a direct comparison of transcript levels of intracellular *MtbΔwhiB3* relative to intracellular wt *Mtb*. Functional group categories are depicted by the colored segments and annotated on the outer rim of the plot. Sub-categorization is annotated on the inside of the plot. See S4 Table.

Using methods developed for microarray analysis of intracellular *Mtb* [22], transcript levels of intracellular *Mtb* and *MtbΔwhiB3* 24 h post-infection of bone-marrow derived macrophages (BMDM) were compared to extracellular controls to reveal relative changes in gene expression in response to the phagosomal environment (Fig 3A and S3 Table). Secondly, we directly compared the transcript levels of intracellular *Mtb* and *MtbΔwhiB3* 24 h post-infection of BMDM (Fig 4 and S4 Table). Distinct gene expression profiles were noted in bacilli from resting versus activated macrophages, consistent with previous reports [23]. However, analysis of the data indicated that the macrophage activation status did not significantly affect differential regulation of genes to the extent of that observed between *Mtb* and *MtbΔwhiB3* under our experimental conditions (Fig 3B), thus we excluded the effect of macrophage activation status. Furthermore, Rohde et al. [22] found that WhiB3 was induced by a decrease in phagosomal pH, which occurs when macrophages are activated by IFN-γ or lipopolysaccharide, or when resting macrophages are infected with *Mtb*.

In the first approach, we observed differential regulation of 333 genes following invasion of BMDM that was significantly dependent on WhiB3 (S3 Table). We found a striking influence of WhiB3 loss on genes involved in intermediary metabolism and respiration (62 genes) in addition to genes implicated in the cell wall and cell processes, such as cell wall transporters (60 genes) (Fig 3A) pointing to the roles of WhiB3 in the adaptation of *Mtb* to the phagosome.

Genes involved in glycolysis (*pgk*), the citric acid cycle (*gltA2*), methyl citrate cycle *(rv1130*, *prpD*), gluconeogenesis (*pckA*), and respiration (*ndh*) were all upregulated in *MtbΔwhiB3*. Genes involved in amino acid metabolism, in particular for His and Leu were upregulated in *MtbΔwhiB3*, while genes involved in the sulphur metabolism (including cysteine metabolism, *cysa2*, *metK*, *rv1464*) were downregulated. While *mutA* was upregulated in the methyl malonyl pathway, genes encoding *cobS* and *cobU* in the biosynthesis of Vitamin B12 (a cofactor for *mutA/B*) were downregulated in *MtbΔwhiB3*. This data suggests WhiB3, which is induced by phagosomal acidification [22], regulates metabolism upon infection to enable *Mtb* to adapt to the intraphagosomal environment. Evidence for the role of WhiB3 in maintaining an energized membrane potential necessary for bioenergetic homeostasis is the *whiB3*-dependent expression of a large number of cell wall ion transporter genes: the magnesium and cobalt transport protein (*corA*) and molybdate permease (*modB*), which are both upregulated in *MtbΔwhiB3*; transporting ATPases, including cation (*ctpG* which is upregulated and *ctpD* which is downregulated in *MtbΔwhiB3*), anion (*rv3680*, which is downregulated in *MtbΔwhiB3*), potassium (*kdpB*) and magnesium (*mgtC*), which are both upregulated in *MtbΔwhiB3*. Other transporters modulated by WhiB3 included ABC transporters (*rv1348*), and transport system proteins for peptides (*dppA*, *dppC*), dicarboxylate (*ktpG*), sugar (*uspC*) and phosphate (*phoY1*). The differential regulation of these cell wall transporters also indicates the role of WhiB3 in response to phagosome acidification and maintaining intrabacterial pH.

WhiB3 also controls the transcription of 25 genes involved in lipid metabolism (Fig 3A) in response to phagosomal cues, notably, those involved in polyketide synthesis, *pks2* and *pks3* were downregulated while *pks5* was upregulated, in addition to genes implicated in lipid degradation (*fadA3* and *fadD5* were downregulated, *fadD8* and *fadE5* were upregulated), mycolic acid metabolism (*acpM*, *fbpB*, *inhA*, *mmaA4* were all upregulated) and mycobactin synthesis (*mbtB*, *mbtC*, *mbt1* were all upregulated.). WhiB3-regulated expression of genes involved in lipid metabolism suggests WhiB3 is involved in altering the cell wall composition in response to the hostile phagosomal environment as well as preparing for the establishment of a persistent infection aided by these lipid virulence factors.

Additionally, a large number of genes involved in redox homeostasis, including oxidoreductases, dehydrogenases, monooxygenases and genes involved in sulphur metabolism were differentially transcribed confirming the role of WhiB3 in maintaining intracellular redox balance.

Previous transcriptomic studies investigating the role of WhiB3 in acidic pH (4.5) resistance of *Mtb* to elucidate mechanisms *Mtb* uses to survive in the phagosomes during immune activation [20] revealed similar overlaps to our intraphagosomal transcriptomic data in the classes of genes regulated by WhiB3, namely, redox homeostasis, amino acid metabolism and lipid metabolism. However, among the genes significantly differentially regulated in these classes, there were only 6 genes in common between the two transcriptomic studies. Whereas genes involved in secretion featured in the acidic transcriptomic data, genes involved in cell wall transporters were regulated by WhiB3 in our intraphagosomal transcriptomic data.

In the second analysis, 121 genes were upregulated and 101 downregulated in intracellular *MtbΔwhiB3* on comparison to intracellular *Mtb* (S4 Table). Genes involved in intermediary metabolism, respiration, the cell wall and cell processes were the most markedly influenced by WhiB3 (Fig 4) demonstrating that WhiB3 is involved in regulating these pathways to protect *Mtb* and potentially alter the hostile environment in the phagosome. Genes involved in the TCA Cycle (*gltA2*, *sucC*) and amino acids feeding into the TCA cycle (*glnE*, *argC*, *argJ*), Vitamin B12 biosynthesis, a cofactor for the methylmalonyl pathway (*cobU*), respiration (*ctaD*, *menB*, *hemZ*), glycolysis (*pgmA*) and its link to glycerol metabolism (*glpD2*) were downregulated in *MtbΔwhiB3* in the phagosome. In contrast, genes involved in nucleotide and deoxyribonucleotide catabolism (*deoC*) were upregulated in *MtbΔwhiB3* to produce glyceraldehyde-3-phosphate for glycolysis. Changes in the central carbon metabolism of the mutant mycobacterium suggest that WhiB3 is pivotal in altering metabolism to ensure intracellular survival.

Genes encoding cell wall transporters (*ctpA* and *fecB*) and enzymes involved in cell wall remodeling (*cut3*, *cut4*, *rv1984c*) were downregulated in *MtbΔwhiB3* indicating that WhiB3 is involved in the continual adjusting of the cell wall to the changing intraphagosomal environment.

Twenty-four genes involved in lipid metabolism were also regulated by WhiB3 in the intraphagosomal environment. Of importance, genes involved in polyketide synthesis (*pks2*, *pks3*, *pks4*, *papA3*) and sulfolipid-1 (*papA1*) and methoxymycolic acid synthesis (*mmA2*) were downregulated in *MtbΔwhiB3*.

Taken together, the intracellular expression data demonstrate that WhiB3 plays a role in the continuous adaptation of *Mtb* to the phagosome environment by adjusting respiration, central carbon metabolism and transporters in the cell wall together with cell wall remodeling. Importantly, as previously observed [9], WhiB3 also plays a role in virulence by regulating the synthesis of immunomodulatory polyketides in macrophages.

### WhiB3 regulates host cell cycle

**Fig 5:**
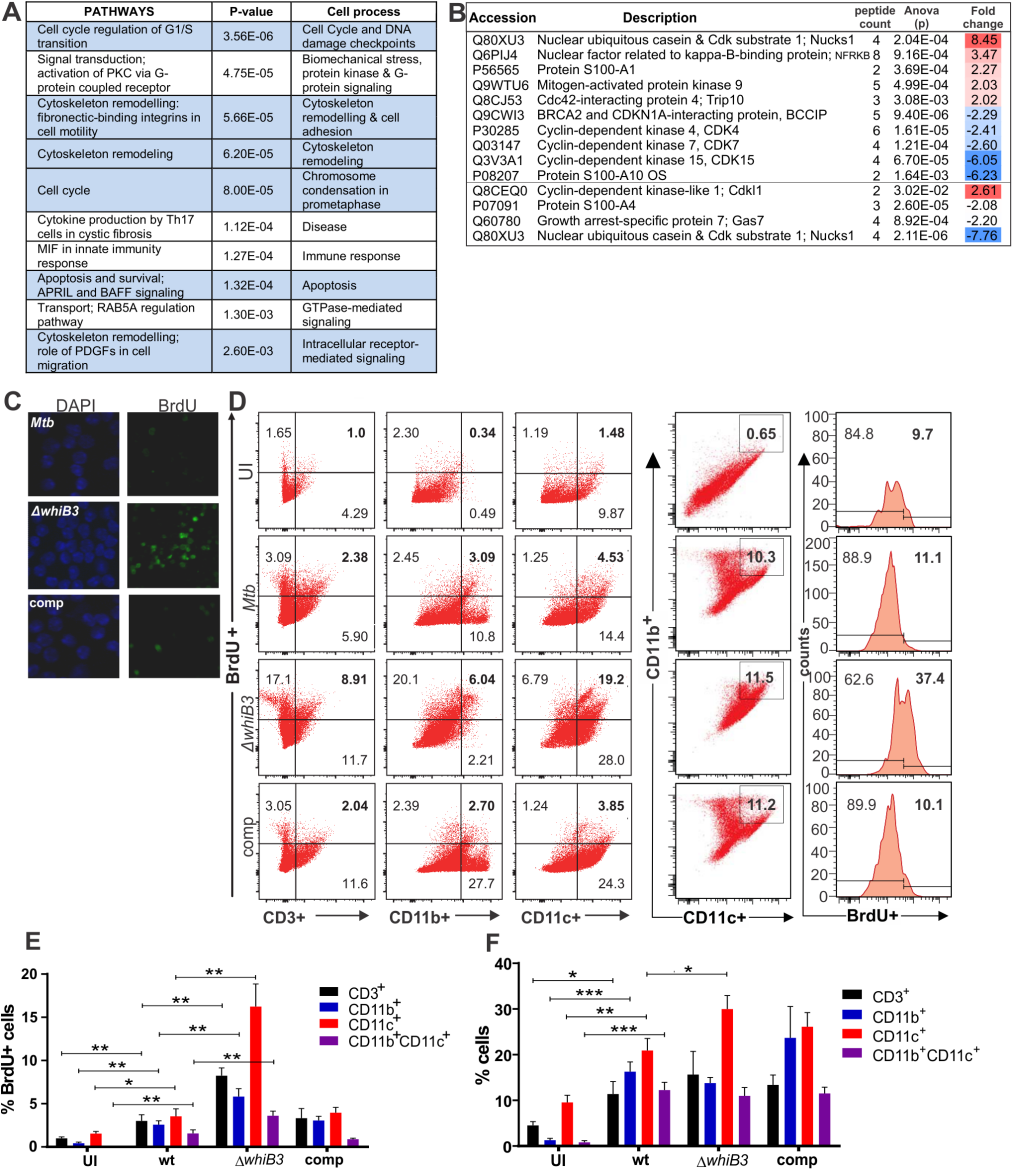
Pathway mapping of *Mtb* infected macrophages reveals that *Mtb* WhiB3 regulates the host cell cycle. **(A)** Top ten host pathways with the most significant differential regulation in *MtbΔwhiB3* infected RAW264.7 macrophages relative to wt *Mtb* H37Rv infected macrophages (p values were calculated with *t* tests). See S2 Fig and S5 and S6 Tables. **(B)** LC-MS/MS identification and quantification of cytoplasmic proteins with more than 2-fold differential expression (p<0.03, ANOVA) in *MtbΔwhiB3* infected RAW264.7 macrophages relative to wt *Mtb* H37Rv infected macrophages. Fold changes were calculated from the means of samples in triplicate. See S7 Table. **(C)** DNA synthesis in RAW264.7 macrophages infected with wt *Mtb* H37Rv, *MtbΔwhiB3* and the complemented (comp) strain (MOI 5) for 24 h. Cells were stained with DAPI (blue) to identify the nuclei and BrdU was added to the cells and incorporated into newly synthesized DNA. **(D)** Representative dot plots of BrdU incorporation versus surface staining with anti-CD3^+^ (lymphocyte marker), anti-CD11b^+^ and anti-CD11c^+^ (myeloid lineage markers) of lung cells isolated from mice infected with wt *Mtb*, *MtbΔwhiB3* or the complemented strain for 6 weeks. CD11b^+^CD11c^+^ double positive cells were also gated and evaluated for BrdU incorporation. **(E)** Percentage of CD3^+^, CD11b^+^, CD11c^+^ or CD11b^+^CD11c^+^ cells isolated from mouse lungs infected with wt *Mtb*, *MtbΔwhiB3* or the complemented strain that are positive for BrdU incorporation. (**F**) Percentage of total CD3^+^, CD11b^+^, CD11c^+^ or CD11b^+^CD11c^+^ cells isolated from mouse lungs infected with wt *Mtb*, *MtbΔwhiB3* or the complemented strain. Data are representative of two independent experiments with three mice per group and were analyzed using the unpaired Student’s *t* test. For the comparison of *MtbΔwhiB3* infected mice versus any of the other mouse groups: * p<0.01.

To gain insights into how the activity of WhiB3 might impact host cell function, we compared the global transcription profiles of replicating RAW264.7 macrophages infected with *Mtb* or *MtbΔwhiB3*. Using Affymetrix Mouse 430 2.0 arrays, 45 037 probe sets were examined of which 629 host genes were differentially regulated by *Mtb* versus *MtbΔwhiB3* (S5 and S6 Tables). To elucidate the biological significance of these genes, we made use of MetaCore^TM^ and identified the top 10 significant host pathways that are differentially regulated in *MtbΔwhiB3* infected macrophages, of which seven are involved in the host cell cycle: including: DNA damage check points, biomechanical stress, cytoskeletal remodeling, chromosome condensation and apoptosis (Fig 5A). RAW264.7 macrophages are capable of proliferation with a doubling time of 11 hours and hence a complete cell cycle. Subsets of differentially regulated critical genes involved in four pathways are listed in S1 Fig. S2 Fig generated by MetaCore^TM^, illustrates the most significant pathways under WhiB3 control involved in the cell cycle regulation of G_1_/S transition and cytoskeletal rearrangement. These gene network findings were supported by subsequent LC-MS/MS proteomic analyses of uninfected, *Mtb* and *MtbΔwhiB3* infected RAW264.7 macrophages, which also revealed differential regulation of proteins involved with the cell cycle between uninfected and *Mtb* or *MtbΔwhiB3* infected macrophages (Fig 5B and S7 Table). In *Mtb* infected macrophages, proteins such as: nuclear ubiquitous casein and Cdk substrate 1 (NUCKS1) [24] and nuclear factor related to kappa-B-binding protein (NFRKB) [25], which are involved in genomic stability, homologous recombination and the DNA repair pathway; the protein S100-A1, a calcium binding protein involved in inhibition of microtubule assembly [26]; mitogen activated protein kinase 9 (MAPK9, also called JNK2) involved in regulating the exit from G_1_ of the cell cycle [27, 28]; Cdc42-interacting protein 4 (Trip10) that promotes cell death or survival in a cell-dependent manner [29], were upregulated when compared to *MtbΔwhiB3* infected macrophages. These proteins are all involved in retaining the cells in the G_0_-G_1_ phase of the cell cycle. Conversely, proteins upregulated in *MtbΔwhiB3* infected macrophages when compared to *Mtb* infected macrophages, include cyclin dependent kinase 4 (CDK4) that initiates the cell cycle through the G_1_-S transition [30], CDK7, which activates CDK4 [31]; protein S100A4 that drives cells into the G_2_/M phase; proteins that attenuate apoptosis and promote proliferation, such as CDK15 [32] and protein S100A10 [33, 34]; and BRACA2 and CDKN1A-interaction protein (BCCIP), which has paradoxical roles in that it has been shown to delay G_1_-S progression but is also a perquisite for proliferation [35, 36]. NUCKS is probably downregulated in the *Mtb* infected cells after 48 hours as DNA repair proteins are not only essential for maintenance of genomic integrity, but often fundamental in DNA replication and mitosis [35] that is possibly being inhibited by the *Mtb* infection. The roles of the upregulation of the cyclin-dependent kinase-like 1 and the downregulation of growth arrest-specific protein 7 (Gas7) in *Mtb* infected macrophages are unclear at this stage. In summary, both transcriptomic and proteomic analysis of infected RAW264.7 macrophages implicate WhiB3 in regulating the host cell cycle.

Based on transcription profiles in S5 and S6 Tables, we hypothesize that factors controlled by *Mtb* WhiB3 will affect host cell DNA synthesis, proliferation and differentiation as the host cell cycle controls the stages at which these events occur. WhiB3 modulation of macrophage DNA synthesis in the cycling mouse macrophage cell line, RAW264.7, was confirmed by fluorescence microscopy of macrophages infected with *Mtb*, *MtbΔwhiB3* and complemented strains for 24 h in the presence of 5-bromo-2'-deoxyuridine (BrdU), a thymidine analogue that is incorporated into DNA during replication and detected with an anti-BrdU antibody. The data revealed greater BrdU incorporation and thus increased DNA synthesis in *MtbΔwhiB3* infected RAW264.7 macrophages than in macrophages infected with wt and complemented strains (Fig 5C). As macrophage proliferation contributes to normal tissue homeostasis [3] and is present at sites of inflammation [4], we investigated the effect of *MtbΔwhiB3* infection on immune cell proliferation *in vivo*. We used BrdU incorporation assays to assess DNA synthesis, leading to host cell proliferation in *Mtb*, *MtbΔwhiB3* and complemented infected mice (Fig 5D–5F). After the mouse lungs were harvested, the single cell suspension was labelled with anti-CD3 (lymphocyte marker), anti-CD11b and anti-CD11c (both myeloid cell markers) and anti-BrdU. Flow cytometry revealed a significantly higher number of CD3^+^, CD11b^+^ or CD11c^+^ cells were positive for BrdU incorporation in the mice infected with *MtbΔwhiB3* than the mice infected with wt or the complemented strain, demonstrating increased DNA synthesis in host immune cells (Fig 5E). Furthermore, the immune cell markers revealed a significantly increased number of total CD11c+ cells in the lungs of mice infected with *MtbΔwhiB3*, indicating proliferation in the mouse lungs infected with *MtbΔwhiB3* (Fig 5F). This increased CD11c^+^ cell proliferation in the *MtbΔwhiB3* infected lung was significantly greater than the proliferation observed in the CD11c^+^ cells in the lungs of mice infected with wt. This suggests that *Mtb* WhiB3 is involved in modulating the natural proliferative immune response in mice that was observed when infected with *MtbΔwhiB3*. In conclusion, these findings propose that *Mtb* WhiB3 plays a role in immunomodulation by suppressing DNA synthesis and CD11c^+^ cell proliferation.

### WhiB3 inhibits transition from G_1_ to the S phase of the host cell cycle

**Fig 6:**
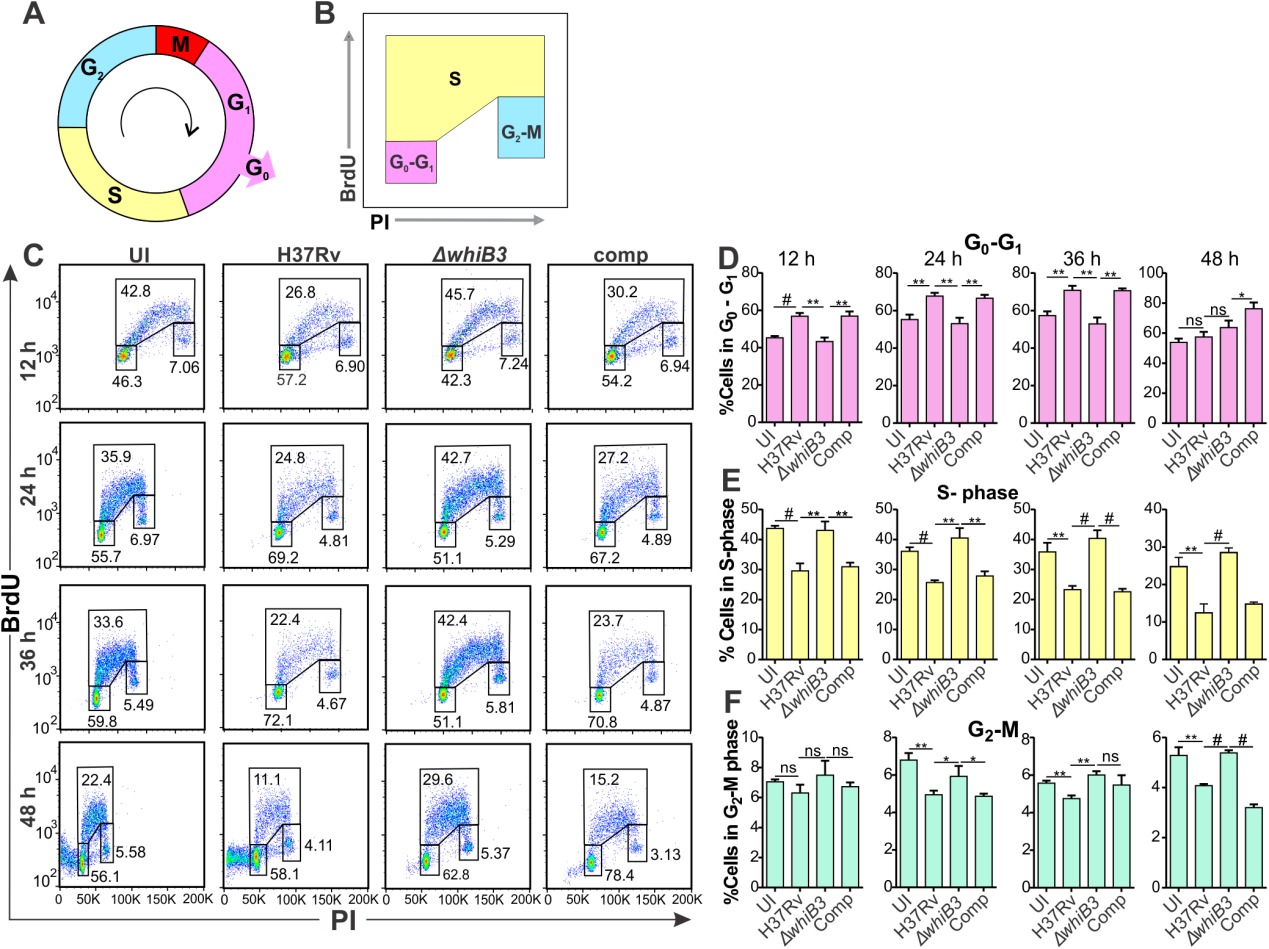
*Mtb* WhiB3 modulates the host cell cycle. **(A)** Schematic diagram of the host cell cycle. **(B)** Schematic diagram of the dot plot of BrdU versus PI indicating the gating strategy. **(C)** Representative dot plots of BrdU and PI incorporation into RAW264.7 macrophages infected with wt *Mtb* H37Rv, *MtbΔwhiB3* or the complemented strain at MOI 5 for the indicated time periods. **(D-F)** Percentage of RAW264.7 cells infected with wt *Mtb*, *MtbΔwhiB3* or the complemented strain at MOI 5 for the indicated time points in the **(D)** G_0_-G_1_ phase, **(E)** S phase and **(F)** G_2_-M phases of the macrophage cell cycle. Error bars represent SD of the mean of triplicate experiments. Data are representative of two independent experiments. Unpaired Student’s t-Test was used to calculate the p value. #, p<0.0001; **, p≤0.001 and * p≤0.05.

Eukaryotic cell division proceeds through a regulated cell cycle comprising of five phases: G_0_, the resting phase, G_1_ (the normal growth phase), S (DNA replication phase), G_2_ (growth and preparing for mitosis), and the M (mitosis) phase (Fig 6A), of which the DNA content can be accurately determined using propidium iodide (PI), and flow cytometry [37]. BrdU incorporation in addition to PI staining emphasizes new DNA synthesis and gives a more improved representation of the S phase (Fig 6B). RAW264.7 macrophages have been used in previous studies to examine cell cycle modulation induced by pathogen infection [38, 39], and we used these macrophages to generate the transcriptomic data (Fig 5A). Thus, for correlation purposes, cell cycle progression in uninfected and *Mtb*, *MtbΔwhiB3* or complemented strain infected macrophages was monitored using PI staining and BrdU incorporation at 12, 24, 36 and 48 h post-infection (Fig 6C). Notably, in comparison to uninfected macrophages, there was a significant increase in *Mtb* infected macrophages in the G_0_/G_1_ phase at 24 and 36 h (Fig 6D), followed by a significant reduction of cells in the S phase (Fig 6E) as well as in the G_2_ phase (Fig 6F) at all time points monitored. Similar trends were observed in the macrophages infected with the complemented strain. This suggests that *Mtb* is prolonging the G_1_ phase of the infected macrophages and reducing their entry into the S-phase. This confirms the host transcriptomic data where the cell cycle regulation of G_1_/S transition is the most differentially regulated pathway (Fig 5A). In addition, the cell cycle of macrophages infected with *MtbΔwhiB3* resembles that of the uninfected cells at all time points investigated (Fig 6D, 6E and 6F), suggesting that the host cell cycle was not significantly affected by the *MtbΔwhiB3* infection. In sum, these findings suggest that *Mtb* inhibits the G_1_/S transition in the macrophage cell cycle and WhiB3 is involved in this modulation.

### Lipids and polyketides under WhiB3 control regulate the host cell cycle

**Fig 7:**
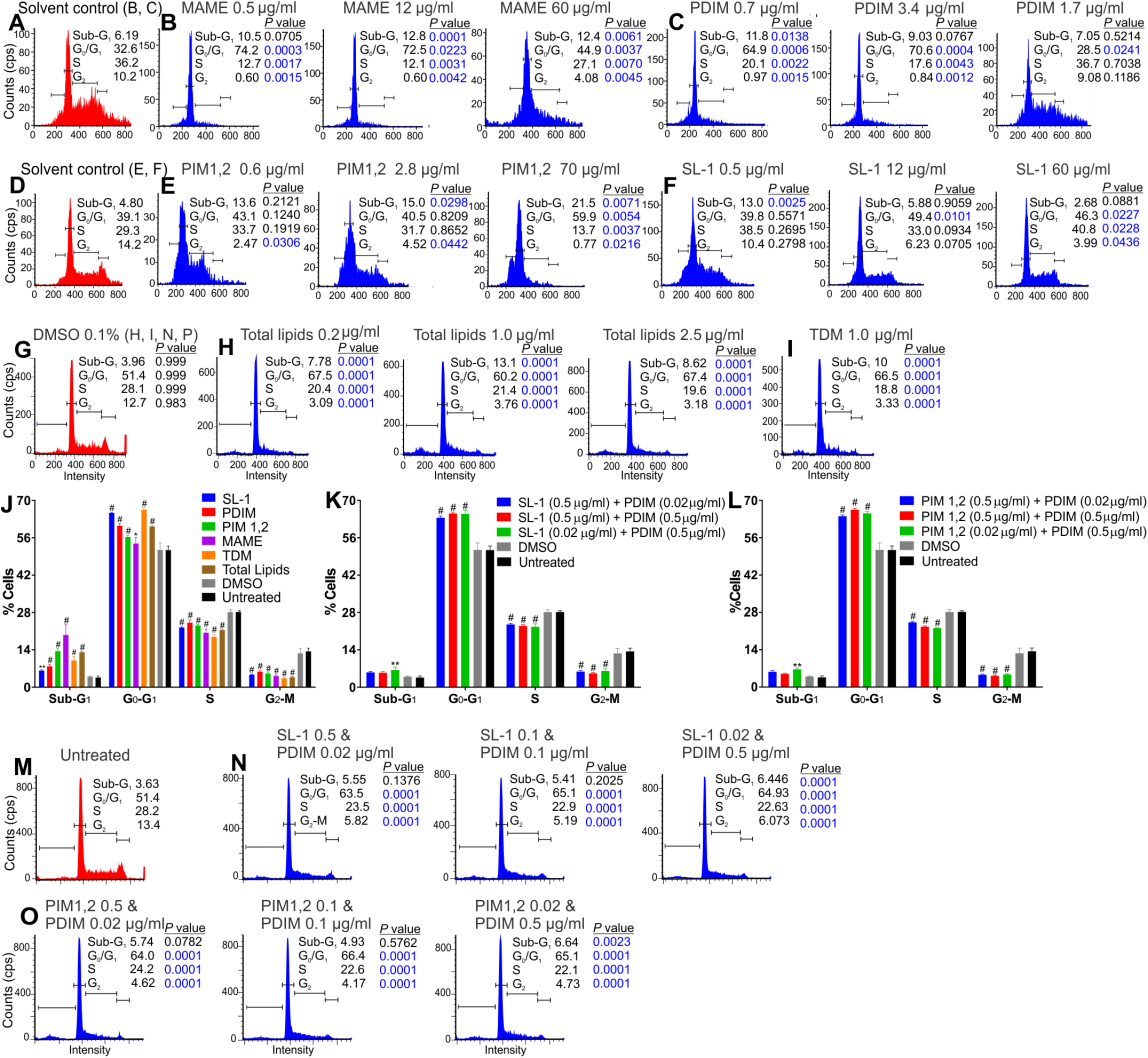
*Mtb* polyketides and cell wall surface molecules under WhiB3 control modulate the host cell cycle. RAW264.7 macrophages **(M**; untreated) were treated in triplicate with **(B)** MAME, **(C)** PDIM, **(E)** PIM 1,2, **(F)** SL-1, **(H)** *Mtb* total lipid extract, or **(I)** TDM at the concentrations indicated for 24 h prior to cell cycle analysis. RAW264.7 cells treated with the solvents in which the lipids were dissolved **(A, D, G)** were used as controls. **(J)** Percentage of RAW264.7 cells in the sub-G_0_, G_0_-G_1_, S and G_2_-M phases of the cell cycle following treatment with 1 μg/ml SL-1, PDIM, PIM 1,2, MAME, TDM, total lipids or vehicle (0.1% DMSO) for 24 h, **(K, N)** combinations of SL-1 and PDIM at the concentrations indicated for 24 h and **(O, L)** combinations of PIM 1,2 and PDIM at the indicated concentrations for 24 h. Error bars represent SD of the mean of triplicate experiments. Data are representative of two independent experiments. Unpaired Student’s t-Test was used to calculate the p value. #, p<0.0001; **, p≤0.005 and *, p≤0.05.

Here, we hypothesize that mycobacterial polyketides and cell surface lipids, some under WhiB3 control, regulate the host cell cycle. Hence, we examined the effect of purified extracts of polyketides and cell surface lipids on the RAW264.7 macrophage cell cycle (Untreated, Fig 7M). The effects of the solvents used to dissolve the lipids on the macrophage cell cycle were assessed and regarded as controls (Fig 7A, 7D and 7G). Of the lipids examined (Fig 7B, 7C, 7E, 7F, 7H and 7I), mycolic acid methyl esters (MAME) had the most potent effect on the host cell cycle. MAME, PDIM-1, PIM 1,2, total lipids and TDM (Fig 7B, 7C, 7E, 7H and 7I) significantly enhanced the percentage of macrophages in the G_1_ phase (p≤0.005) together with significant reductions of that in the S and G_2_ phases. Higher concentrations of PDIM (Fig 7C) did not have significant effects (p>0.05) on the host cell cycle, probably due to aggregation of PDIM. PIM 1,2 (Fig 7E) also significantly increased the percentage of apoptotic cells, indicated by the sub-G_1_ population (p<0.01). Lower concentrations of PIM 1,2 (Fig 7E) significantly lowered cell numbers in the G_2_ phase with no significant effects on the G_1_ and S-phases. SL-1 (Fig 7F) modulated the host cell cycle with significant increments of cells in the G_1_ and S phases, and a significant decrease of cells in the G_2_ phase (p<0.05). Lower concentrations of SL-1 (Fig 7F) significantly increased the number of apoptotic cells (p<0.005). When the effects of the polyketides and lipids at identical concentrations, 1 μg/ml, on RAW264.7 macrophages over 24 h were compared, all the lipids examined induced a significant increase in the number of cells in the Sub-G_1_ (apoptotic cells) and G_0_-G_1_ phase, with a significant concomitant decrease in percentage of cells in the S phase and the G_2_ phase when compared to the untreated cells and vehicle (0.1% DMSO)-treated cells (Fig 7J). In a physiological setting, these lipids would be present in combinations, hence we examined the cell cycles of macrophages treated with a combination of SL-1 and PDIM, and a combination PIM1,2 and PDIM for 24 h in opposing and equal concentrations (Fig 7K, 7L, 7N and 7O). Regardless of the varied concentrations of the lipids examined, both combinations resulted in significant increases in the percentage of cells in the G_0_-G_1_ phase and significant reductions in the number of cells in the S and G_2_-M phases suggesting that the combination of lipids also prohibited the macrophage cell cycle transition from the G_1_ to the S phase. In sum, we found that mycobacterial lipids do modulate the RAW264.7 macrophage cell cycle by inhibiting the G_1_-S transition.

### Genetic evidence of polyketides under WhiB3 control dysregulating the host cell cycle

**Fig 8:**
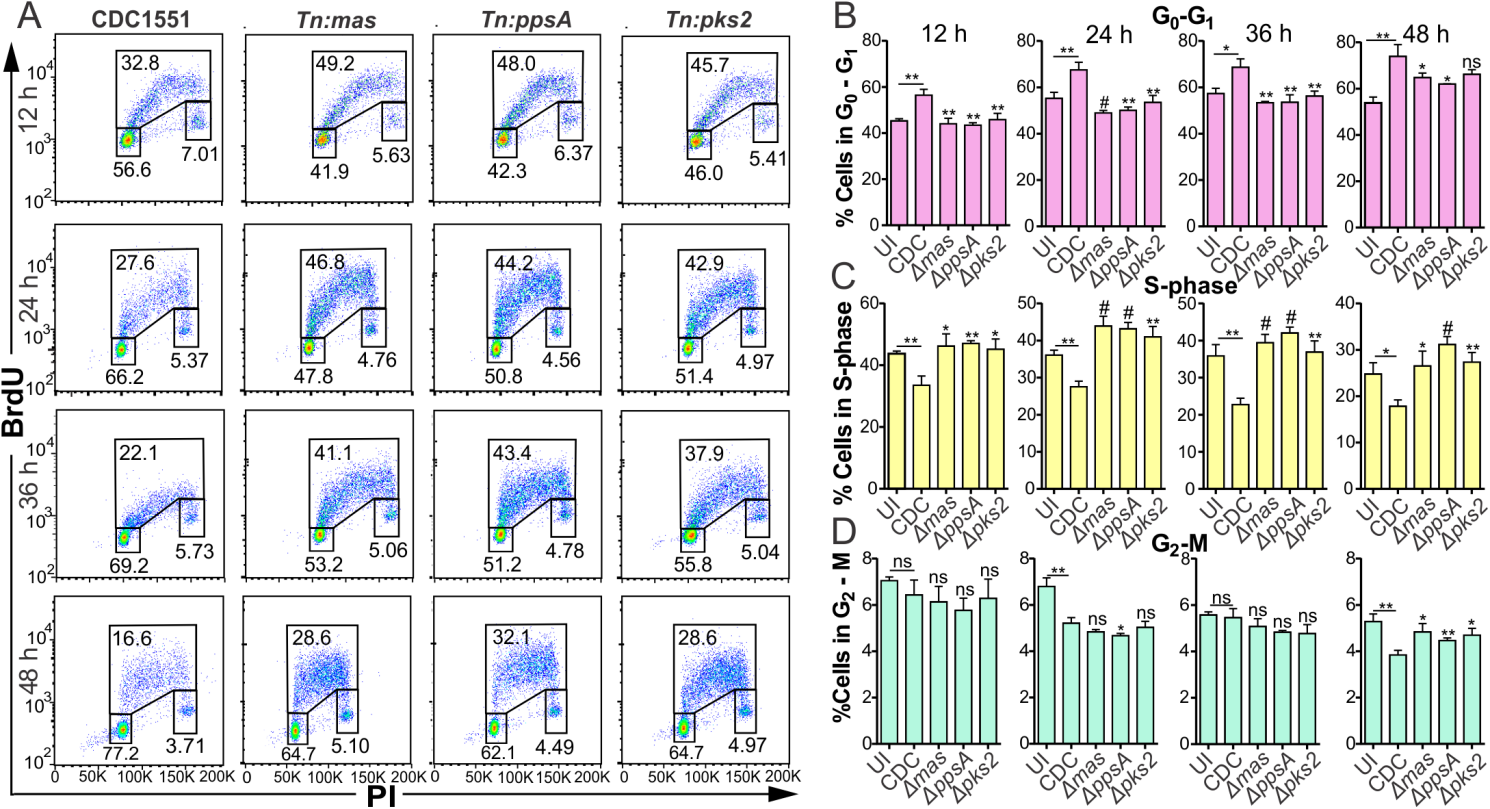
Infection with *Mtb* polyketide mutants do not alter the macrophage cell cycle. **(A)** Representative dot plots of BrdU and PI incorporation into RAW264.7 macrophages infected with wt *Mtb*, *Mtb Tn*:*pks2*, *Mtb Tn*:*mas* or *Mtb Tn*:*ppsA* at MOI 5 for the indicated time points. **(B-D)** Percentage of RAW264.7 cells infected with wt *Mtb* CDC1551, *Mtb Tn*:*pks2*, *Mtb Tn*:*mas* or *Mtb Tn*:*ppsA*, at MOI 5 for the indicated time points in the **(B)** G_0_-G_1_ phase, **(C)** S phase and **(D)** G_2_-M phases of the macrophage cell cycle. Error bars represent SD from the mean of triplicate experiments. Data are representative of two independent experiments. Unpaired Student’s *t* Test was used to calculate the p value. #, p≤0.0001; **, p≤0.001; *, p≤0.05; symbols above the bars indicates comparison to macrophages infected with wt *Mtb*.

To further validate our findings that mycobacterial lipids under WhiB3 control modulated the host cell cycle, we investigated if the infection of RAW264.7 macrophages with *Mtb* mutants for polyketides under WhiB3 control demonstrated similar alterations of the host cell cycle as *MtbΔwhiB3*. Macrophages were infected with *Mtb*, *Mtb Tn*:*pks2* (necessary for SL-1 production), *Mtb Tn*:*mas* and *Mtb Tn*:*ppsA* (both required for PDIM production) for 12, 24, 36 and 48 h. Using BrdU and PI incorporation (Fig 8A), we found that macrophages infected with *Mtb Tn*:*pks2*, *Mtb Tn*:*mas* or *Mtb Tn*:*ppsA* had significantly greater numbers of cells in the S-phase (Fig 8C), with concomitant increments of cells in the G_2_-M phase (Fig 8D) and fewer cells in the G_0_-G_1_ phase (Fig 8B) at all time points investigated in comparison to that in wt infected macrophages. These cell cycle modulations are similar to the trends in the host cell cycle observed in uninfected and *MtbΔwhiB3* infected macrophages (Fig 6D, 6E and 6F).

Thus, the parallels observed in the host cell cycle progression between macrophages infected with the *whiB3* mutant and those infected with the polyketide mutants strongly suggests that the polyketides under WhiB3 control are responsible for altering the host cell cycle.

## Discussion

A major challenge in the TB field is to understand the precise mechanism of disease to develop novel therapeutic strategies against *Mtb* pathogenesis. *Mtb* WhiB3 has emerged as a model virulence redox regulator, although the exact mechanism is unknown. Initially, we investigated mechanisms of WhiB3 virulence using transcriptomic analysis of *Mtb* and infected macrophages. *In vitro* and intraphagosomal *Mtb* microarrays indicated the WhiB3 dependence of bioenergetic metabolism and its role in enabling *Mtb* to adapt to the phagosomal environment by cell wall remodeling and metabolic modulation, in particular lipid metabolism, amino acid metabolism, redox and sulfur metabolism (Figs 3 and 4). Unexpectedly, the host microarray data revealed that *Mtb* WhiB3 differentially regulated host genes involved in the host cell cycle and DNA damage checkpoints in macrophages that are capable of cell division (Fig 5). Although there is the supposition that macrophages are terminally differentiated, there is clear evidence for *in vivo* lung macrophage proliferation in the lung [1], which is the site of infection in pulmonary TB. Thus, the proliferating lung macrophages will be predisposed to modulation by *Mtb* lipids and cell wall components after ingestion of *Mtb*. We subsequently hypothesized that *Mtb* WhiB3 maintains bioenergetic homeostasis to control production of factors that subvert host cell cycle. Extracellular flux analysis of *Mtb* strains provided further evidence that WhiB3 regulates *Mtb* bioenergetics in response to available energy sources. Previously, we have demonstrated that WhiB3 maintains redox homeostasis by regulating virulence lipid production in *Mtb* [9]. As redox homeostasis is closely linked to bioenergetic homeostasis due to electron flow through the critical redox centers in the electron transport chain, the controlled generation of superoxide and hydrogen peroxide from the respiratory chain, and NO modulation of respiration, it can be inferred that bioenergetic homeostasis is essential for virulence lipid production. Using BrdU incorporation, we demonstrated increased DNA synthesis in lymphocyte and myeloid cells, and increased proliferation of CD11c^+^ cells in lungs of mice infected with *MtbΔwhiB3* than in those from mice infected with *Mtb* (Fig 5E and 5F). Cell cycle analysis revealed that infection with *Mtb* inhibited the G_1_/S transition in macrophages when compared to uninfected macrophages. WhiB3, PDIM and SL-1 have proven necessary for this G_1_/S block as infection with *MtbΔwhiB3* or SL-1 or PDIM mutants did not appear to affect the host cycle. As treatment of macrophages with purified polyketides and other *Mtb* surface lipids also arrested macrophages in the G_0_/G_1_ phase, we proposed that polyketides under WhiB3 control are responsible for the *Mtb* modulation of the host cell cycle. Collectively, our findings discover a novel mechanism whereby *Mtb* modulates the immune system by altering the host cell cycle to promote long-term persistence. Since the cell cycle is an attractive drug target for several diseases, this new knowledge could serve as the foundation for new host-directed therapeutic drug discovery efforts.

Previously, we have shown that there is an accumulation of PDIM along with reduction in SL-1 in the WhiB3 mutant both *in vitro* and in macrophages [9] pointing towards a pleiotropic phenotype. *M*. *ulcerans* produces a known cyclomodulin, the polyketide-derived macrolide mycolactone, which was found to induce G_0_/G_1_ cell cycle arrest in L929 mouse fibroblast cells [13]. We found that infection with *Mtb* inhibited the G_0_/G_1_ transition in the macrophage cell cycle when compared to uninfected replicating macrophages (Fig 6D, 6E and 6F). *M*. *bovis* BCG infection of bronchial airway epithelial cells has also been reported to induce cell cycle arrest at the G_0_-G_1_ phase [40]. Investigations to ascertain if polyketides and lipids, some under WhiB3 control, had similar cyclomodulatory effects as mycolactone disclosed that MAME, PDIM, PIM 1,2, TDM and total lipid extracts from *Mtb* did indeed arrest macrophages in the G_0_/G_1_ of the cell cycle (Fig 7A–7I). As combinations of the polyketides are more probable *in vivo*, we also examined combinations of SL-1 together with PDIM and PIM 1,2 together with PDIM and found that both combinations inhibited the transition of the macrophage cell cycle from the G_1_ to the S phase (Fig 7N and 7O). PIM 1,2 also induced apoptosis in the macrophages, similar to that observed in L929 cells and J774 mouse macrophages after 3–5 days of exposure to mycolactone [41]. This underscores the significance of the timing and period of exposure to these virulence factors, thus emphasizing the importance of WhiB3 control of virulence factors in response to the host environment (such as O_2_, NO and carbon sources). Macrophages infected with *Mtb* mutants of polyketides (SL-1 and PDIM) under WhiB3 control displayed similar trends in their cell cycle to that observed in uninfected macrophages (Fig 8A–8D) much like the cell cycle in *MtbΔwhiB3* infected cells (Fig 6C–6F). This proposes that polyketides under WhiB3 control have cyclomodulatory effects on the host cell cycle.

What is the advantage of *Mtb* interfering with the host cell cycle? A unique characteristic of most pathogens producing cyclomodulins is their ability to establish long, persistent infections. In particular, cyclomodulins such as VacA, CDT and mycolactone have immunomodulatory properties at least *in vitro*. *Mtb* results in a long, persistent infection and *Mtb* polyketides and lipids that we have implicated as cyclomodulins also have immunomodulatory effects. PIMs and lipoarabinomannans from *Mtb* have been reported to elicit chemokine and cytokine responses from mononuclear cells [42, 43] and bacterial lipids that are released from intracellular bacteria induce cytokines, such as IL-6, that suppress T-cell proliferation [44]. Furthermore, PIMs and TDM exert granulomatous responses in mice [45–47]. Mycolic acid and PDIM resist degradation and accumulate in chronically infected tissues [48, 49], thereby exerting long-term effects at the site of infection [50, 51] likely redirecting the host cell cycle of specific cell types to establish persistent infection. Previously, it has been found that lipids shed by intracellular mycobacteria, such as TDM and PIM2, spread via endocytic network throughout the macrophage, and via exocytic vesicles to neighboring uninfected cells [52], thus possibly altering the cell cycles of neighboring uninfected macrophages. Furthermore, it has been demonstrated that *Mtb* infects airway epithelial cells [53, 54] and microfold cells [55], both capable of replication, suggesting that their cell cycle and replication may also be modulated by lipids shed by the intracellular mycobacteria. Limitations of our studies include using purified lipid preparations at artificial concentrations and combinations and *in vitro* observations of their cyclomodulatory effects. Naturally, different proportions of a mixture of lipids will be produced *in vivo* to modulate the host.

Additional immunomodulatory effects are demonstrated by our *in vivo* studies, where we observed increased DNA synthesis in the lung lymphoid and myeloid cells and greater proliferation of lung CD11c^+^ cells in mice infected with *MtbΔwhiB3* than that in *Mtb* infected and uninfected mice (Fig 5E and 5F). Infection with *MtbΔwhiB3* does not appear to affect the host cell cycle and likely induces an appropriate immune response that contains the infection and promotes survival of the mice. This is corroborated by longer survival times of mice infected with the *whiB3* mutant in comparison to mice infected with wt *Mtb* that was previously observed [15]. In contrast, wt *Mtb* induces a G_0_/G_1_ cell cycle arrest in the macrophages. This probably enables the mycobacteria to subvert innate killing mechanisms and evade the adaptive immune system as non-cycling cells are less likely to be killed by cytotoxic T cells [56]. These immunoevasion strategies likely reduce the priming of the immune system as evidenced by the lower CD11c+ cell proliferation and reduced DNA synthesis in the lymphoid and myeloid cells observed in the lungs of the *Mtb* infected mice and allows the bacilli to establish a persistent infection resulting in shorter mouse survival times [15]. In the case of the uninfected mice, no proliferation was observed as the immune system is not primed due to a lack of infection.

**Fig 9:**
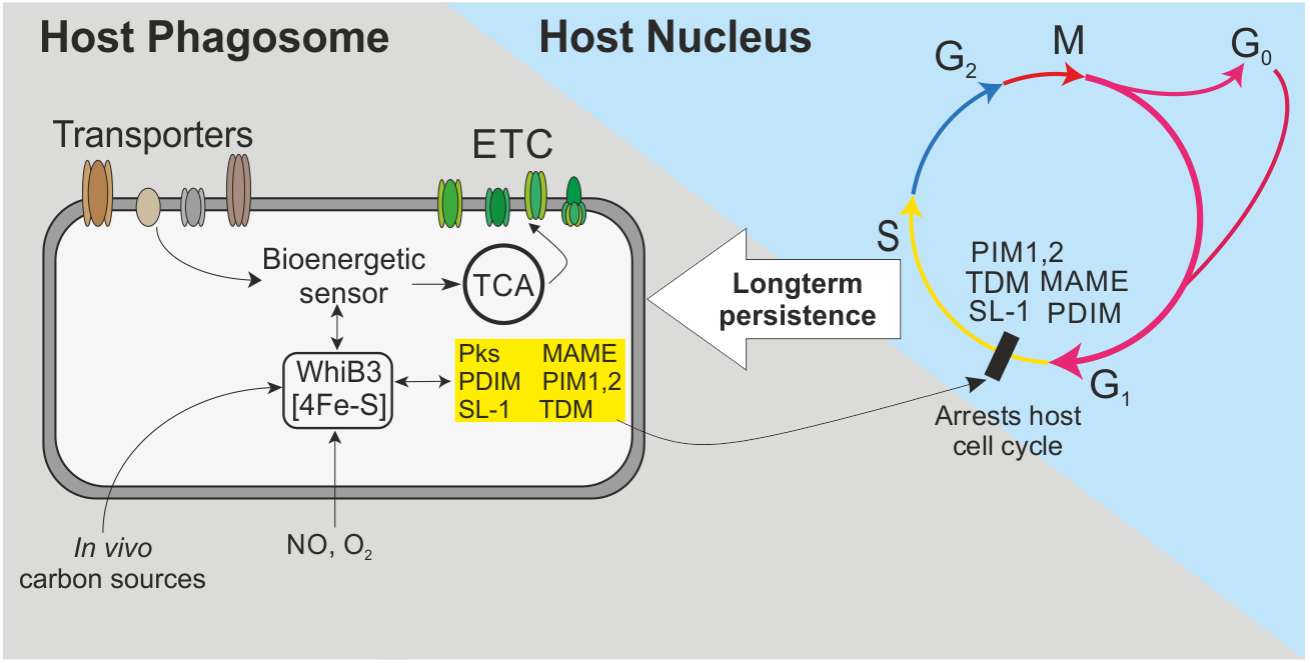
Model whereby *Mtb* arrests the macrophage cell cycle to establish long term infection. *Mtb* WhiB3 is a redox sensor that senses NO and low levels of oxygen (hypoxia) along with *in vivo* carbon sources available and accordingly modulates bioenergetic metabolism in response to the immediate environment. Bioenergetic homeostasis is essential for the transcription and production of polyketides under WhiB3 control. Lipids and polyketides are released from *Mtb* into the infected macrophage, where they arrest the host’s cell cycle and modulate the immune response to establish a persistent infection.

What are the plausible mechanisms by which WhiB3 regulates the cyclomodulins? The WhiB3 4Fe-4S cluster is sensitive to NO and O_2_ [16] that are implicated in dormancy, and O_2_-mediated Fe-S cluster degradation and oxidation of apo-WhiB3 affects DNA binding, which controls the production of cyclomodulins. Since O_2_ gradients exist in the lung and TB granuloma, O_2_ levels, and hence respiration, may synchronize polyketide and lipid production to colonize the host. This is likely as it has been demonstrated that in response to O_2_ gradients *in vivo*, *Shigella* respiration coordinates type III secretion systems via the 4Fe-4S cluster protein FNR [57], which is also degraded by O_2_ and regulates DNA-binding. Respiration implicates energy metabolism in the synchronization of lipid metabolism. This is supported by our bioenergetic data, which demonstrates that when *Mtb* is subjected to bioenergetic stress, WhiB3 regulates OXPHOS and carbon catabolism in response to the available carbon sources. This mechanism has *in vivo* relevance since it suggests a role for WhiB3 in regulating the timing and levels of polyketide and lipid production during distinct stages of disease in response to changing carbon sources, bioenergetic and redox stresses. These include environmental dormancy signals such as hypoxia, NO levels and changing fatty acid substrates (Fig 9).

In conclusion, our findings suggest that bioenergetic homeostasis is an essential component of *Mtb* pathogenesis. The capacity of WhiB3 to control polyketide and lipid cyclomodulins in response to *in vivo* signals intimates that either acute infection or persistence can be achieved by manipulating the host cell cycle. The concept that *Mtb* can modulate the immune system by arresting the host cell cycle has therapeutic implications and establishes a new paradigm for understanding *Mtb* persistence.

## Methods

### Strains and culturing

*Mtb* H37Rv, *MtbΔwhiB3*, and *MtbΔwhiB3* complemented strain, *Mtb* CDC1551, *Mtb Tn*:*pks2*, *Mtb Tn*:*mas* and *Mtb Tn*:*ppsA* were grown shaking at 37°C in 7H9 broth (Difco) and 0.1% Tween 80 in inkwell bottles or 7H11 agar (Difco) supplemented with 0.2% glycerol, Middlebrook albumin-dextrose-catalase (ADC) enrichment. Anhydrous tetracycline was added to a final concentration of 100 ng/ml to induce expression of WhiB3 in the *MtbΔwhiB3* complemented strain culture. The same amount of anhydrous tetracycline was added to the cultures of the wt *Mtb* and *MtbΔwhiB3* strains for the metabolic flux bioenergetic assays [16]. Bone marrow-derived macrophages were isolated from C57BL/6 mice and together with the mouse macrophage RAW264.7 cells (ATCC TIB-71, Lot No. 61524889, tested for mycoplasma using LookOut Mycoplasma PCR Detection kit, Sigma), were maintained in Dulbecco’s modified Eagle’s medium (DMEM, Sigma) supplemented with 10% FCS, 1% sodium pyruvate, 1% L- glutamine, 1% penicillin and streptomycin and 20% L cell-conditioned medium in the case of the bone marrow-derived macrophages. Media lacking antibiotics was added to the macrophages 24 h before infection.

### *In vitro* microarray hybridization and data analysis

RNA was obtained from three biological replicates of exponentially growing (OD600 = 0.7) *Mtb* H37Rv (wild type, wt) and *MtbΔwhiB3* strains cultured in 7H9 medium supplemented with ADST using RNApro, and was subsequently treated with DNase to remove any residual genomic DNA. The quality of RNA was examined using the nano-drop and by running the samples on Experion gene chips (BioRad). Expression analyses were performed by the Center for Applied Genomics (www.cag.icph.org) at the Public Health Research Institute (PHRI) as previously described [58]. These microarrays consist of 4,295 70-mer oligonucleotides representing 3,924 open reading frames (ORFs) from *Mtb* strain H37Rv (http://www.sanger.ac.uk). The complete gene list and array layout can be found at www.cag.icph.org/downloads_page.htm). A total of 0.5 to 1 μg of total RNA from each sample was used for each microarray with three biological replicates for each strain. The detailed labeling and hybridization protocol can be obtained at www.cag.icph.org/downloads_page.htm. Total RNA was prepared in triplicate and used for cDNA synthesis, which was performed using random primers and labeled with cyanine-3 or cyanine-5 dUTP (Perkin Elmer to generate cyanine 3 or cyanine 5 dUTP labeled probes. Hybridization was performed overnight. For each pair of samples in each experiment, dye flips were performed. After washing, the arrays were dried by centrifugation (100 x *g*, 2 min) and scanned using GenePix4000B scanner (Molecular Devices). The images were processed using GenePix 5.1 software. Data were filtered by removing all spots that were below the background noise or flagged as ‘bad’. The chips were normalized by the print-tip Lowess method. The ratio of the mean median intensity of Cy5 over the mean median intensity of Cy3 was determined for each spot and the fold change values were calculated. We only accepted genes, which were up-regulated by at least 1.75-fold, with a *q* value of 1%. The *q* value is the equivalent of the *p* value after multiple-testing correction. Functional classification and pathway annotations were performed using TubercuList dataset (http://genolist.pasteur.fr/TubercuList) and the KEGG database.

### Q-PCR analysis

Cells were harvested from two biological replicates of all *Mtb* strains and RNA was isolated as described previously [58]. First-strand synthesis was performed by using 500 ng total RNA with iScript Select cDNA synthesis kit (Bio-Rad) using random oligonucleotides. PCR was performed in three technical replicates using gene specific primers. Expression of genes was analyzed with real-time PCR using iQ SYBR Green supermix (Bio-Rad) and a BioRad iCycler 5 with an iQ Multicolour Real-Time PCR Detection System (Bio-Rad). PCR efficiencies were normalized to obtain accurate expression levels. For comparisons between wt *Mtb* and *MtbΔWhiB3*, the induction ratio for each gene was normalized to *Mtb* 16s rRNA expression.

### Intraphagosomal microarray

Total RNA was isolated from intraphagosomal wt *Mtb* H37Rv and *MtbΔwhiB3* in infected naïve or immune-activated mouse bone marrow derived macrophages, amplified, labeled and analyzed as previously described [22]. Activated macrophages (MØ) were primed with 100 units/ml of recombinant mouse interferon-ϒ (Peprotech) for 24 h followed by pre-activation with 10 ng/ml of LPS (Sigma) for 16 h. MØ were infected (MOI ~4:1) with *Mtb* H37Rv or *MtbΔwhiB3* grown to mid-log phase (OD = 0.6–0.8) in standing 75 cm^2^ vented tissue culture flasks. Prior to infections, bacteria were declumped by 10 passages through a 21 gauge needle in PBS + 0.01% Tween-80 and diluted in 1 ml of uptake buffer (PBS with 4.5 mg/ml glucose, 5 mg/ml defatted BSA, and 1 mg/ml gelatin) and 5 ml binding medium (DMEM with 5% fetal calf serum, 10 mM HEPES, pH 7.4). After 4 hr infection, extracellular bacteria were removed and replaced with fresh macrophage medium without antibiotics. At 24 h post-infection, addition of GTC lysis buffer (4 M guanidine thiocyanate, 0.5% Na N-lauryl sarcosine, 25 mM sodium citrate, and 0.1 M β-mercaptoethanol) selectively lysed MØ, halted RNA transcription/degradation while leaving mycobacteria intact as previously described. Samples were vortexed and passed five times through a 21G needle to shear bone marrow derived macrophages and reduce viscosity. Intracellular mycobacteria were recovered by centrifugation at 3500 x *g* for 30 min. Pelleted *Mtb* were lysed in 65°C Trizol using a BeadBeater and 0.1 mm silicon beads. Total RNA was isolated from Trizol lysates by chloroform extraction and Qiagen RNeasy column purification and treated with Turbo DNAfree DNase (Ambion) to remove residual DNA contamination. 250 ng of total RNA was linearly amplified using the MessageAmpTM-II Bacteria RNA Amplification system (Ambion), during which amino-allyl UTP was incorporated into RNA products to allow post-labeling with Alexa fluorophores. Amino-allyl modified aRNA were labeled with Alexa Fluor 555 (extracellular control) and Alexa Fluor 647 (intramacrophage) (Invitrogen) and hybridized to custom oligonucleotide arrays as described previously [22, 59, 60]. For each condition (strain + macrophage), probes from intracellular samples were hybridized against probes derived from the extracellular control of the same strain in a two-color format.

Oligonucleotide microarrays were used that consisted of 4295 ORF-specific 70-mers (Operon) representing 3924 ORFs from strain H37Rv plus 371 ORFs from strain CDC1551 with <97% homology to corresponding H37Rv genes. Oligos were spotted in duplicate on Corning UltraGAP (amino-silane coated) slides. The microarray platform used can be accessed via NCBI’s Gene Expression Omnibu (GEO) database under platform accession number GPL5754.

Microarrays were analyzed with a GenePix 4000B scanner (Axon Instruments, Inc.) with preliminary image analysis, spot intensity determination, background measurements, spot quality assessment and flagging conducted with Imagene software (version 6.0, Biodiscovery). Poor quality spots with signal intensities less than three standard deviations above background were excluded from further analysis. Subsequent normalization, statistical analysis, and visualization of array data were performed with Genespring 7.3 (Agilent). Genes with significant changes in expression levels relative to controls were identified based on fold change and reproducibility between replicates (p-value <0.05). One-way ANOVA was used to identify genes with significant differences in expression between conditions. Where indicated, multiple testing corrections (Benjamini and Hochberg false discovery rate (FDR)) were applied.

### Host microarray

Total RNA were isolated from three biological replicates of RAW264.7 cells infected with wt *Mtb*, *MtbΔwhiB3* or complemented strains using the NuGEN Ovation RNA Amplification System V2 in combination with the FL-Ovation cDNA Biotin Module V2 were carried out in accordance with the guidelines detailed in the corresponding NuGEN technical manuals. Total RNA was amplified using the NuGEN Ovation RNA Amplification System V2. First-strand synthesis of cDNA was performed using a unique first-strand DNA/RNA chimeric primer mix, resulting in cDNA/mRNA hybrid molecules. Following fragmentation of the mRNA component of the cDNA/mRNA molecules, second-strand synthesis was performed and double-stranded cDNA was formed with a unique DNA/RNA heteroduplex at one end. In the final amplification step, RNA within the heteroduplex was degraded using RNaseH, and replication of the resultant single-stranded cDNA was achieved through DNA/RNA chimeric primer binding and DNA polymerase enzymatic activity. The amplified single-stranded cDNA was purified for accurate quantitation of the cDNA and to ensure optimal performance during the fragmentation and labeling process. The single stranded cDNA was assessed using spectrophotometric methods in combination with the Agilent Bioanalyzer. The appropriate amount of amplified single-stranded cDNA was fragmented and labeled using the FL-Ovation cDNA Biotin Module V2. The enzymatically and chemically fragmented product (50–100 nt) was labeled via the attachment of biotinylated nucleotides onto the 3'-end of the fragmented cDNA. The resultant fragmented and labeled cDNA was added to the hybridization cocktail in accordance with the NuGEN guidelines for hybridization onto Affymetrix GeneChip arrays. Following the hybridization for 16–18 h at 45°C in an Affymetrix GeneChip Hybridization Oven 640, the array was washed and stained on the GeneChip Fluidics Station 450 using the appropriate fluidics script, before being inserted into the Affymetrix autoloader carousel and scanned using the GeneChip Scanner 3000. Pathway analysis was performed by Almac Diagnostics using Meta Core (Thomson Reuters).

### Host microarray data analysis

The Rosetta Error Model pipelines have been applied to the raw data for background correction, data summarization and normalization. Cross-array scaling was applied to normalize the sample data. Genes with low intensity values that are close to background were filtered out. The standard deviation of the average background (σ) intensity is applied to estimate the background. Less Stringent (Intensity Threshold; 2 Std Background [4.807], Intensity p-value < 0.05, number of genes, 25858) and Stringent groups (Intensity Threshold; 3 Std Background [7.211], Intensity p-value < 0.01, number of genes, 23128) were identified (see S5 and S6 Tables). Statistical parameters used include: t-tests and fold cut-off (2-fold up-regulated or down-regulated); significant p-value (<0.05 with Multiple Testing Correction [Benjamini & Hochberg False Discovery Rate] for stringent genes.

### ATP quantification

Intracellular ATP was quantified by using the bioluminescence based Enliten ATP assay system (Promega). Duplicate aliquots of *Mtb* mycobacteria (100 μl) were collected at various time points and immediately heat-inactivated. Cell lysates (25 μl) were transferred into 96-well white plates and the assay was performed as per manufacturer’s instructions. The emitted luminescence was detected with a Synergy Hybrid Reader (Biotek Instruments) and was expressed as relative luminescence units. ATP standards were included in all experiments as internal controls.

### Metabolic flux bioenergetic assays

*Mtb* bacilli were washed and resuspended in unbuffered Dulbecco Modified Eagle’s medium (DMEM, Sigma) that was supplemented with 1.85 g/l NaCl, 2 g/l glucose, 2 ml/l glycerol, and 2 mM L-glutamine and the pH was adjusted to 7.35–7.40. *Mtb* bacilli were seeded into XF96 cell culture microplates (Seahorse Biosciences, MA, USA) that were coated with Cell-Tak (BD Biosciences) at a density of 2x10^6^ cells/well and adhered to the bottom of XF96 cell culture microtiter plates through centrifugation. OCR and ECAR were measured [21] before and after the addition of an uncoupler, carbonyl cyanide m-chlorophenyl hydrazone (CCCP) to a final concentration of 3.0 μM using a XF96 Extracellular Flux Analyser from Seahorse Biosciences (Billerica, MA, USA) according to manufacturer’s instructions. Three biological replicates were performed, each in triplicate (technical).

### Confocal microscopy

RAW264.7 macrophages were seeded onto glass coverslips and infected with wt *Mtb*, *MtbΔWhiB3* and the complemented strain at an MOI 5 for 24 h. Cells were washed twice with prewarmed phosphate-buffered saline, pH 7.4 (PBS) prior to fixing the cells in 3.7% formaldehyde solution in PBS for 10 minutes. After washing the cells twice with PBS, the fixed cells were treated with a solution of acetone at -20°C or 0.1% Triton X-100 in PBS for 3 to 5 minutes. The cells were washed twice with PBS again followed by incubating the fixed cells with PBS containing 1% bovine serum albumin for 20–30 min to reduce background staining. The fixed cells were then stained with DAPI and BrdU (Life Technologies) according to manufacturing instructions and viewed with a 63x objective on a Leica SP1 UV Confocal Laser Scanning Microscope.

### LC-MS/MS determination of NADH/NAD^+^ ratios in *Mtb* lysates

*Mtb* H37Rv cultures were cultured in triplicate in various carbon sources (50 μM cholesterol, palmitate, propionate, glucose, acetate) and no carbon sources till an OD_600_ of 0.8. At the specific time points 1.8 ml of each culture was pelleted in triplicate, 1 ml of cold chloroform:methanol at a ratio of 2:1, (containing the internal standard, 6-amininicotinic acid) was added and stored at -80°C until further processing. An additional 1 ml of each culture was pelleted and formalin fixed for weight determination. During further processing the bacilli lysate chloroform:methanol mixture was spun through a 0.22 μm filter and the filtrate evaporated under reduced pressure, while centrifuged. The dried pellet was reconstituted in 200 μl water overnight and submitted for LC-MS/MS analysis. The NADH/NAD^+^ ratios were determined with two different LC-MS/MS methods. For the NADH/NAD^+^ ratios the analytes were separated on a Zorbax Eclipse Plus C18 column (2.1 x 100 mm, 3.5 μm) using a gradient elution program at a flow rate of 200 μl/min and an injection volume of 10 μl. Two mobile phases were used: mobile phase A (A, 0.1% formic acid in water) and mobile phase B (B, 0.1% formic acid in acetonitrile). The gradient program was as follows: 0–5 minutes, a linear increase from 0–90% of B; 5–15 minutes, 90% of B; 15–15.1 minutes, 0% of B; and 15.1–27 minutes, 0% of B. For NADH the precursor ion monitored in positive mode was 666 *m/z* with the two product ions being 649 *m/z* and 107.8 *m/z*, for NAD^+^ 664 *m/z* was monitored with the two product ions 427.9 *m/z* and 135.9 *m/z*, respectively. This experiment was performed twice using three biological replicates.

### Monitoring DNA synthesis *in vivo*

*Mus musculus* Balb/c female mice (8 weeks old) were infected with low dose (~200 CFU per lung on Day 1) of wt *Mtb*, *MtbΔWhiB3* or complemented strains via the aerosol route. After 6 weeks, 3 mice from each group were injected intraperitoneally with BrdU (100 μl of 10 mg/ml) 24 h prior to being sacrificed. After the lungs were harvested, they were sliced into small pieces and washed with RPMI 1640 media. The lung tissue was then digested with 0.5 mg/ml type IV collagenase A (Roche) and 40 units/ml DNAse 1 (Roche) to isolate interstitial cells. Tissue was mechanically dissociated with a GentleMacs system (Lung program, Militenyi) followed by incubation at 37°C for 60 min and a second round of dissociation. Cells were then washed twice in medium and strained through a 70 μm cell strainer. Isolated lung cells were surface stained with mAbs (BD Biosciences) directed against CD3 (PE-Cy7; 17A2), CD11b (BV605; M1/70) and CD11c (PerCP-Cy5.5; HL3) at 4°C for 30 min followed by two washes with PBS. After surface staining, these cells were fixed with 250 μl of fixation buffer (BD Bioscience) for 30 min on ice and permeabilized with 1 x permeabilization buffer (BD Biosciences). Cells were labelled with APC-conjugated anti-BrdU antibody (Bu20a; Biolegend) for 1 h on ice. Cells were washed by centrifugation. Data acquisition was performed on a BD Biosciences Fortessa flow cytometer and analysis was performed with Flowjo Vx.0.7. Two independent experiments were performed using three mice per *Mtb* strain in each experiment.

### Cell cycle analysis

RAW264.7 cells were seeded at 0.2 x 10^6^ cells/well in 6-well plates, cultured in DMEM with 10% FBS at 37^°^C for 12 h. After 12 h, cells were infected with *Mtb* strains at MOI 5 and incubated for 12, 24, 36 and 48 h. Prior to harvesting, the cells were pulse labelled with bromodeoxyuridine (BrdU) for 45 min. Uninfected cells and unlabelled BrdU cells were used as negative controls. The harvested cells were stained with the live/dead stain (Ex ~633 nm/ Em ~780 nm, Invitrogen), washed and fixed in ice cold 70% ethanol for 12 h. After washing with PBS, the cells were treated with 2N HCl for 20 min to expose BrdU labeled DNA and the acidic pH was neutralized with 0.1 M sodium borate buffer, pH 9.0. The cells were then permeabilized with 1X permeabilization buffer (BD Biosciences) containing 0.3% Triton X-100 to ensure nuclear membrane permeabilization and washed once with PBS. Cells were labelled with APC-conjugated anti-BrdU antibody (Biolegend) for 1 h on ice. After washing by centrifugation, the cells were resuspended in 0.2 ml of propidium iodide solution (Sigma), and allowed to incubate for 30 min on ice. Cell cycle analysis was performed by data acquisition with Fortessa flow cytometer (BD Biosciences) and analyzed with FlowJo Vx.0.7. Two independent experiments were performed in triplicate.

### Host proteomic analyses

Protein extracts from RAW264.7 cells uninfected and infected with wt *Mtb*, *MtbΔWhiB3* or complemented strains, trypsin digested using Filter Aided sample preparation for cytoplasmic protein extracts [61]. Digested processed protein extracts were analyzed at the Centre for Proteomic and Genomic Research (CPGR) in Cape Town, South Africa using Nano-Reverse Phase LC-MS/MS analysis and the data was evaluated with ProgenesisQI version 1 (Nonlinear Dynamics, UK) as described in S1 Protocol. Three technical replicates were performed for each condition (macrophages uninfected or infected with wt *Mtb*, *MtbΔWhiB3* or complemented strains).

### Ethics statement

Mouse studies were approved by the University of KwaZulu-Natal Animal Research Ethics Committee (Protocol reference number: 125/14/Animal). Balb/c mice were maintained at the Biomedical Resource Centre of University of KwaZulu-Natal (UKZN), Westville and infected mice were maintained at Biosafety level 3 in our facilities at the K-RITH Tower, Nelson R. Mandela School of Medicine, UKZN, Durban, South Africa in accordance with the guidelines set forth by the South African National Standard (SANS 10386:2008).

## Supporting information

S1 Protocol Host proteomic analysis. (DOCX)

S1 Fig Heat maps of selected differentially expressed genes of infected macrophages categorized according to pathways in which they are involved. (PDF)

S2 Fig **Metacore reports of host genes differentially expressed in the pathways involved in (A) cell cycle regulation of the G1/S transition and (B) cytoskeletal remodeling of RAW264.7 macrophages infected with *MtbΔwhiB3* versus *Mtb*.** Thermometer icons in the schematic diagrams beside the gene names give an indication of the extent of upregulation (red) or downregulation (blue).
(PDF)

S1 Table Genes differentially regulated in *MtbΔwhiB3* versus *Mtb* H37Rv *in vitro*. Microarrays were performed on *Mtb****Δ****WhiB3* and *Mtb* H37Rv grown in 7H9 till mid-log phase. Includes genes >1.52 and <0.6 (p<0.05).
(XLSX)

S2 Table Validation of DNA microarray results by qRT-PCR. To confirm the microarray results, mRNA levels of selected genes were compared between *Mtb****Δ****whiB3* and wt *Mtb* using QRT-PCR as described in the Methods.
(XLSX)

S3 Table WhiB3-dependent gene regulation during macrophage infection. Changes in transcript levels of intracellular *Mtb****Δ****whiB3* and wt *Mtb* after 24 hr infection of resting and activated macrophages were determined by normalizing to data from strain-matched extracellular controls. Genes with significantly different induction/repression between the two strains were identified by Volcano plots, using ANOVA p<0.05 and 1.2X fold change as cutoffs. A total of 333 genes were identified that exhibited WhiB3-dependent regulation during macrophage infection. The values in the Ratio column represent wt *Mtb/Mtb****Δ****whiB3*.
(XLSX)

S4 Table Direct comparison of transcript levels of *MtbΔwhiB3* and wt *Mtb*. Array data derived from intracellular samples of *Mtb****Δ****whiB3* and wt *Mtb* were directly compared (i.e. intracellular wt *Mtb* versus intracellular *Mtb****Δ****whiB3*) following median normalization as described previously (Homolka et al. 2010). 235 genes with significantly different transcript levels in macrophages after 24hr infection were identified. Volcano plots, using ANOVA p<0.05 (with Benjamini Hochberg false-discovery rate) and 1.2X fold change as cutoffs.
(XLSX)

S5 Table List of host genes differentially expressed in RAW264.7 cells infected with *Mtb* or *MtbΔwhiB3* based on less stringent criteria. (XLSX)

S6 Table List of host genes differentially expressed in RAW264.7 macrophages infected with *Mtb* or *MtbΔwhiB3* based on stringent criteria. (XLSX)

S7 Table List of proteins involved with the cell cycle and cytoskeleton that are differentially regulated in RAW264.7 cells infected with *MtbΔwhiB3* and wt *Mtb* for 24 and 48 h. (XLSX)
