# Correction: *Mycobacterium tuberculosis* arrests host cycle at the G_1_/S transition to establish long term infection

**DOI:** 10.1371/journal.ppat.1006490

**Published:** 2017-07-14

**Authors:** Bridgette M. Cumming, Md. Aejazur Rahman, Dirk A. Lamprecht, Kyle H. Rohde, Vikram Saini, John H. Adamson, David G. Russell, Adrie J. C. Steyn

In the Funding section, the authors would like to include the following statement: “Open access publication of this article has been made possible through support from the Victor Daitz Information Gateway, an initiative of the Victor Daitz Foundation and the University of KwaZulu-Natal (http://daitzgateway.org/).”
