# Supplementary material for: Mycobacterium tuberculosis arrests host cycle at the G1/S transition to establish long term infection
Source: PLoS Pathog. 2017 May 22;13(5):e1006389. doi: 10.1371/journal.ppat.1006389 (PMC5456404; doi:10.1371/journal.ppat.1006389)
Supplement: S1 Protocol — (DOCX) [file ppat.1006389.s001.docx]

**S1 Protocol. Host Proteomic Analysis**

*Trypsin digestion: Filter Aided Sample Preparation (FASP)* (Wisniewski et al., 2009). All reagents are analytical grade or equivalent. 150 µg of total protein from each lysate (Cytoplasmic) was placed in a 1.5 ml LoBind Eppendorf tube and reduced with 0.1 volume 50 mM triscarboxyethyl phosphine (TCEP; Sigma 646547) for 1 h at 60^o^C. Reduced protein was placed on a 30 kDA MWCO centrifugal filter (Amicon ultra; Millipore UFC503024). Samples were concentrated down to 30 µl by centrifugation at 13000 g. The retentate was alkylated by the addition of 100 µl 15 mM methyl methanethiosulphonate (MMTS; Sigma 208795) in 8 M urea (Sigma U5128) 100 mM triethyl ammonium bicarbonate (TEAB; Sigma 17902) buffer for 15 min at 20^o^C. Buffer was exchanged by adding 350 µl 8 M urea 100 mM TEAB buffer to the alkylated sample and reducing the volume to 30 µl by centrifugation at 13000 g. Buffer exchange was repeated to a total of three times to ensure excess detergent was washed out. Urea concentration was reduced by two washes with 100 µl 100 mM TEAB, each time reducing the retentate volume to 30 µl by centrifugation at 13000 g. Proteins were digested by adding trypsin (Promega PRV5111) in 100 mM TEAB to a final protein:trypsin ratio of 50:1, and then incubated for 18 h at 37^o^C. Peptides were collected in a new collection tube by 13000 g centrifugation, followed by two MilliQ H_2_O filter washes. Samples were dried and re-suspended in 0.05% trifluoroacetic acid (TFA; Sigma T6508), 5% acetonitrile (Burdick and Jackson) in which they were stored until LCMS analysis.

*LC-MS/MS analysis*. Nano-Reverse Phase LC-MS/MS analysis was conducted with a Q-Exactive quadrupole-Orbitrap mass spectrometer (Thermo Fisher Scientific, USA) coupled with a Dionex ultimate 3000 nano-HPLC system (Thermo Fisher Scientific, USA). The mobile phases consisted of solvent A (0.1% formic acid in water) and solvent B (90% ACN, 10% water, and 0.1% formic acid). Tryptic peptides from each sample were dried under vacuum and re-solubilized in sample loading buffer (95% water, 5% Acetonitrile, 0.05% TFA). An estimated 1 µg of total peptide was loaded on a C18 trap column (100 µm×20 mm×5 µm). Chromatographic separation was performed with an Acclaim® PepMap100 C18 column (75 µm×250 mm×2 µm) (Thermo Fisher Scientific, USA). The gradient was delivered at 400 nl/min and consisted of a linear gradient of mobile phase B initiating from solvent B: 6–25% over 82 min. The mass spectrometer was operated in positive ion mode with a capillary temperature of 250°C. The applied electrospray voltage was 1.95 kV. In one cycle the top 12 most abundant peptides were chosen for MS/MS fragmentation with a dynamic exclusion of 30 s. Details of data acquisition parameters are given in Table A.

**Table A. Details of data acquisition parameters for LC-MS/MS for proteomic analysis.**

| Full Scan | |
| --- | --- |
| Resolution | 70,000 (@ m/z 200) |
| AGC target value | 3e6 |
| Scan range | 355-1750 m/z |
| Maximal injection time (ms) | 120 |
| Data-dependent MS/MS | |
| Resolution | 17,500 (@ m/z 200) |
| AGC target value | 1e5 |
| Maximal injection time (ms) | 75 |
| Isolation window width (m/z) | 3 |
| NCE (%) | 26 |
| Data-dependent Settings | |
| Underfill ratio (%) | 1% |
| Charge exclusion | Charge states 1,5-8,>8 |
| Peptide match | preferred |
| Exclusion isotopes | on |
| Dynamic exclusion (s) | 30 |

*Data analysis:* *Label free relative protein quantitation* LC–MS/MS raw data were aligned, normalized and quantified (relative) using ProgenesisQI version 1 (Nonlinear Dynamics, UK). Relative protein quantitation was conducted using non-conflicting peptides. At least 2 unique peptides were used for quantification of proteins. ProgenesisQI aligns chromatograms from different experimental conditions and then determines features in the data (m/z and retention time pairs) that differ across chromatograms. Byonic search results (see below) were exported in XML format and imported into Progenesis to provide the necessary identification information once alignment and frame analysis had been completed. Based on 3 technical replicates per condition a normalized fold change, normalized standard deviation and a normalized p-value (ANOVA) was calculated for each protein. Proteins were considered regulated if their fold change was greater than or equal to 2 and their corresponding p-value (ANOVA) was less than or equal to 0.05. All protein quantification was normalized to total ion current (TIC).

*Protein Identification for label free* quantification The Uniprot mouse reference proteome (dated 22/06/2015) was used as the sequence database for protein identification (http://www.uniprot.org/). Database interrogation was performed with the Byonic algorithm (Proteinmetrics, San Carlos,CA; version PMI-Byonic-Com). Main search parameters are detailed in Table B. Global false discovery rate was controlled to below 1%.

**Table B. Details of the protein search parameters for the proteomic analysis.**

| Type of search | MS/MS Ion Search |
| --- | --- |
| Enzyme | Trypsin, fully specific |
| Fixed modifications | Methylthio / +45.987721 @ C \| fixed |
| Variable modifications | \| Gln->pyro-Glu / -17.026549 @ N-Term Q \| rare1 \| \| --- \| \| Glu->pyro-Glu / -18.010565 @ N-Term E \| rare1 \| \| Oxidation / +15.994915 @ H, M, W \| common1 \| \| Deamidated / +0.984016 @ N, Q \| common1 \| |
| Mass values | Monoisotopic |
| Peptide mass tolerance | 20 ppm |
| Fragment mass tolerance | 20 ppm |
| Max missed cleavages | 2 |
| Fragmentation type | QTOF/HCD |

**Supporting Reference**

Wisniewski, J.R., Zougman, A., Nagaraj, N., and Mann, M. (2009). Universal sample preparation method for proteome analysis. Nat Methods *6*, 359-362.
