# Supplementary figures and images for: Mycobacterium tuberculosis arrests host cycle at the G1/S transition to establish long term infection
Source: PLoS Pathog. 2017 May 22;13(5):e1006389. doi: 10.1371/journal.ppat.1006389 (PMC5456404; doi:10.1371/journal.ppat.1006389)

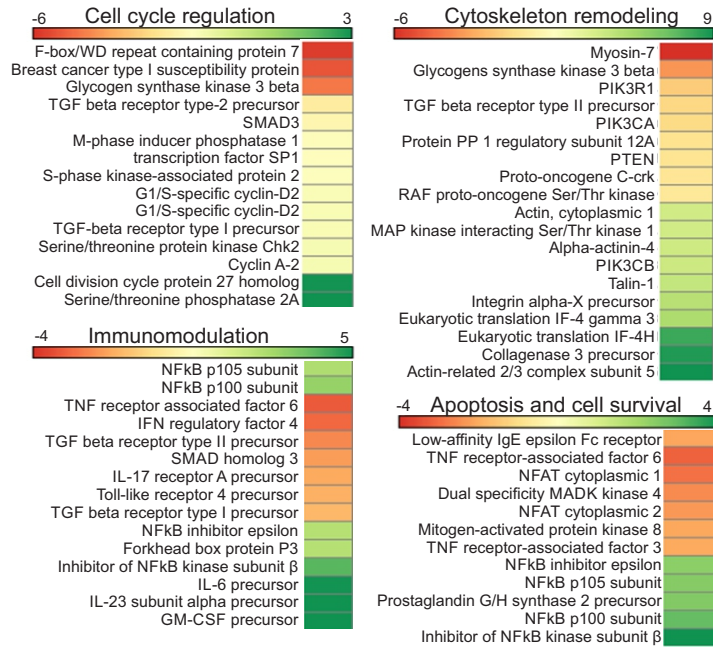

Supplement: S1 Fig — (PDF) [file ppat.1006389.s002.pdf]
