# Supplementary material for: Mycobacterium tuberculosis arrests host cycle at the G1/S transition to establish long term infection
Source: PLoS Pathog. 2017 May 22;13(5):e1006389. doi: 10.1371/journal.ppat.1006389 (PMC5456404; doi:10.1371/journal.ppat.1006389)
Supplement: S2 Fig — Metacore reports of host genes differentially expressed in the pathways involved in (A) cell cycle regulation of the G1/S transition and (B) cytoskeletal remodeling of RAW264.7 macrophages infected with MtbΔwhiB3 versus Mtb. Thermometer icons in the schematic diagrams beside the gene names give an indication of the extent of upregulation (red) or downregulation (blue). (PDF) [file ppat.1006389.s003.pdf]

A. Cell cycle Regulation of G1/S transition

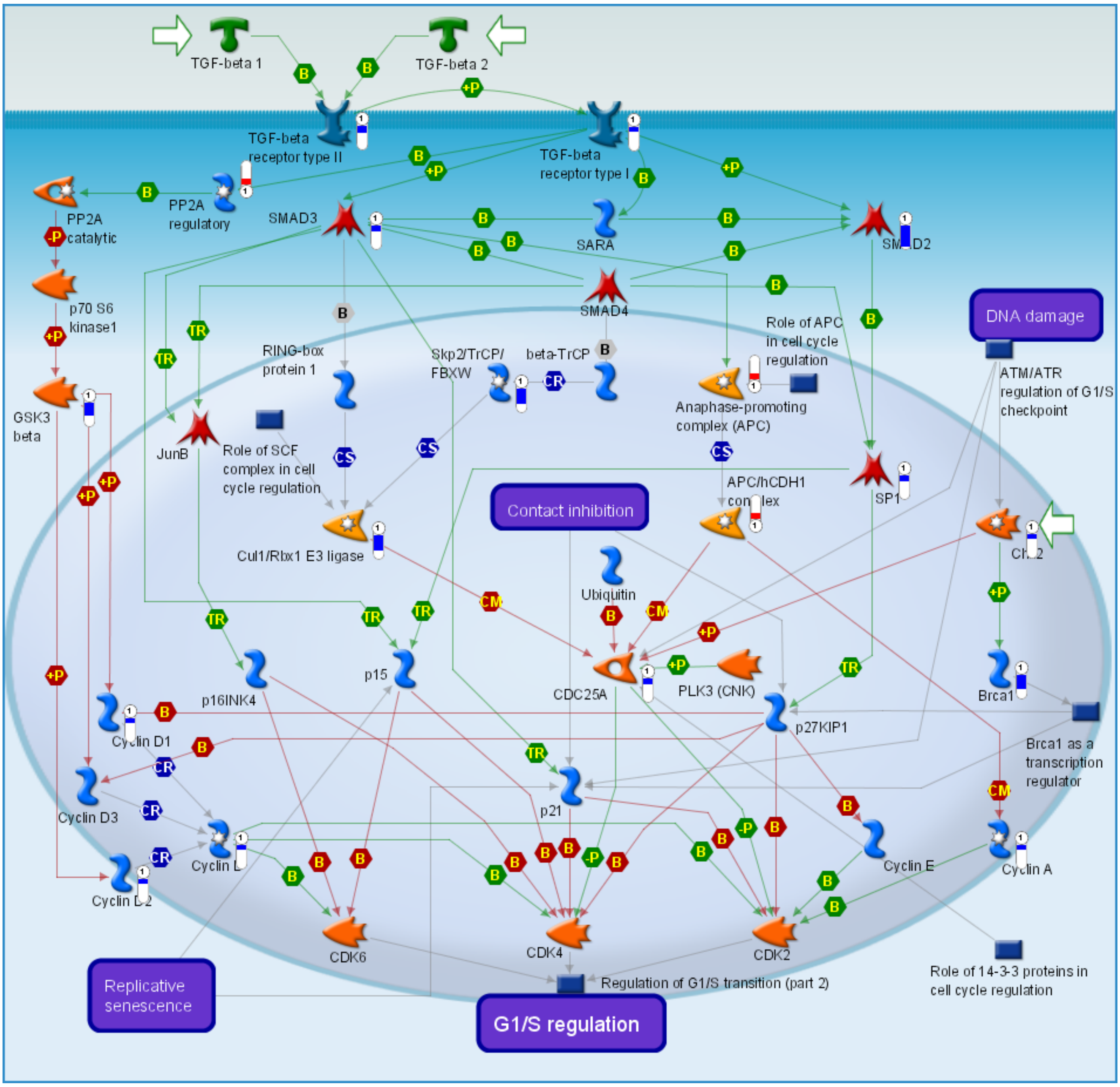

Gene Symbol	Protein	Class	Protein name	Fold Change
ANAPC1	Protein		Anaphase-promoting complex subunit 1	
ANAPC10	APC1_HUMAN		Anaphase-promoting complex subunit 10	
ANAPC11	APC10_HUMAN		Anaphase-promoting complex subunit 11	
ANAPC13	APC11_HUMAN		Anaphase-promoting complex subunit 13	
ANAPC2	APC13_HUMAN		Anaphase-promoting complex subunit 2	

ANAPC4	ANC2_HUMAN	Anaphase-promoting complex subunit 4	
ANAPC5	APC4_HUMAN	Anaphase-promoting complex subunit 5	
ANAPC7	APC5_HUMAN	Anaphase-promoting complex subunit 7	
BRCA1	APC7_HUMAN	Breast cancer type 1 susceptibility protein	-5.38
BTRC	BRCA1_HUMAN	F-box/WD repeat-containing protein 1A	
CCNA1	FBW1A_HUMAN	Cyclin-A1.	
CCNA2	CCNA1_HUMAN	Cyclin-A2	-1.72
CCND1	CCNA2_HUMAN	G1/S-specific cyclin-D1	-1.78
CCND2	CCND1_HUMAN	G1/S-specific cyclin-D2.	-1.79
CCND3	CCND2_HUMAN	G1/S-specific cyclin-D3.	
CCNE1	CCND3_HUMAN	G1/S-specific cyclin-E1.	
CDC16	CCNE1_HUMAN	Cell division cycle protein 16 homolog	
CDC23	CDC16_HUMAN	Cell division cycle protein 23 homolog	
CDC25A	CDC23_HUMAN	M-phase inducer phosphatase 1	-2.02
CDC26	MPIP1_HUMAN	Anaphase-promoting complex subunit CDC26	
CDC27	CDC26_HUMAN	Cell division cycle protein 27 homolog	2.05
CDK2	CDC27_HUMAN	Cell division protein kinase 2	
CDK4	CDK2_HUMAN	Cell division protein kinase 4	
CDK6	CDK4_HUMAN	Cell division protein kinase 6	
CDKN1A	CDK6_HUMAN	Cyclin-dependent kinase inhibitor 1	
CDKN1B	CDN1A_HUMAN	Cyclin-dependent kinase inhibitor 1B	
CDKN2A	CDN1B_HUMAN	Cyclin-dependent kinase inhibitor 2A, isoforms 1/2/3	
	CD2A1_HUMAN	Cyclin-dependent kinase inhibitor 2A, isoform 4	
CDKN2B	CD2A2_HUMAN	Cyclin-dependent kinase 4 inhibitor B	
CHEK2	CDN2B_HUMAN	Serine/threonine-protein kinase Chk2	-1.75
CUL1	CHK2_HUMAN	Cullin-1	
FBXW11	CUL1_HUMAN	F-box/WD repeat-containing protein 11	
FBXW7	FBW1B_HUMAN	F-box/WD repeat-containing protein 7	-5.77

FZR1	FBXW7_HUMAN		Fizzy-related protein homolog	
GSK3B	FZR_HUMAN		Glycogen synthase kinase-3 beta	-4.81
JUNB	GSK3B_HUMAN		Transcription factor jun-B.	
PLK3	JUNB_HUMAN		Serine/threonine-protein kinase PLK3	
PPP2CA	PLK3_HUMAN		Serine/threonine-protein phosphatase 2A catalytic subunit alpha isoform	
PPP2CB	PP2AA_HUMAN		Serine/threonine-protein phosphatase 2A catalytic subunit beta isoform	
PPP2R2A	PP2AB_HUMAN		Serine/threonine-protein phosphatase 2A 55 kDa regulatory subunit B alpha isoform	
PPP2R2B	2ABA_HUMAN		Serine/threonine-protein phosphatase 2A 55 kDa regulatory subunit B beta isoform	
PPP2R2C	2ABB_HUMAN		Serine/threonine-protein phosphatase 2A 55 kDa regulatory subunit B gamma isoform	
PPP2R3A	2ABG_HUMAN		Serine/threonine-protein phosphatase 2A regulatory subunit B'' subunit alpha	
PPP2R3B	P2R3A_HUMAN		Serine/threonine-protein phosphatase 2A regulatory subunit B'' subunit beta	
PPP2R4	P2R3B_HUMAN		Serine/threonine-protein phosphatase 2A regulatory subunit B'	
PPP2R5A	PTPA_HUMAN		Serine/threonine-protein phosphatase 2A 56 kDa regulatory subunit alpha isoform	2.11
PPP2R5B	2A5A_HUMAN		Serine/threonine-protein phosphatase 2A 56 kDa regulatory subunit beta isoform	
PPP2R5C	2A5B_HUMAN		Serine/threonine-protein phosphatase 2A 56 kDa regulatory subunit gamma isoform	
PPP2R5D	2A5G_HUMAN		Serine/threonine-protein phosphatase 2A 56 kDa regulatory subunit delta isoform	
PPP2R5E	2A5D_HUMAN		Serine/threonine-protein phosphatase 2A 56 kDa regulatory subunit epsilon isoform	
RBX1	2A5E_HUMAN		RING-box protein 1	
RPS27A	RBX1_HUMAN		40S ribosomal protein S27a.	
RPS6KB1	RS27A_HUMAN		Ribosomal protein S6 kinase beta-1	
SKP1	KS6B1_HUMAN		S-phase kinase-associated protein 1	
SKP2	SKP1_HUMAN		S-phase kinase-associated protein 2	-1.91

SMAD2	SKP2_HUMAN		Mothers against decapentaplegic homolog 2	-8.03
SMAD3	SMAD2_HUMAN		Mothers against decapentaplegic homolog 3	-2.23
SMAD4	SMAD3_HUMAN		Mothers against decapentaplegic homolog 4	
SP1	SMAD4_HUMAN		Transcription factor Sp1.	-1.97
TGFB1	SP1_HUMAN		Transforming growth factor beta-1 precursor	
TGFB2	TGFB1_HUMAN		Transforming growth factor beta-2 precursor	
TGFBR1	TGFB2_HUMAN		TGF-beta receptor type-1 precursor	-1.76
TGFBR2	TGFR1_HUMAN		TGF-beta receptor type-2 precursor	-2.54
UBA52	TGFR2_HUMAN		Ubiquitin.	
	UBIQ_HUMAN		60S ribosomal protein L40	
UBB	RL40_HUMAN		Ubiquitin.	
ZFYVE9	UBIQ_HUMAN		Zinc finger FYVE domain-containing protein 9	

B [Cytoskeleton remodeling fibronectin-binding integrins in cell motility]

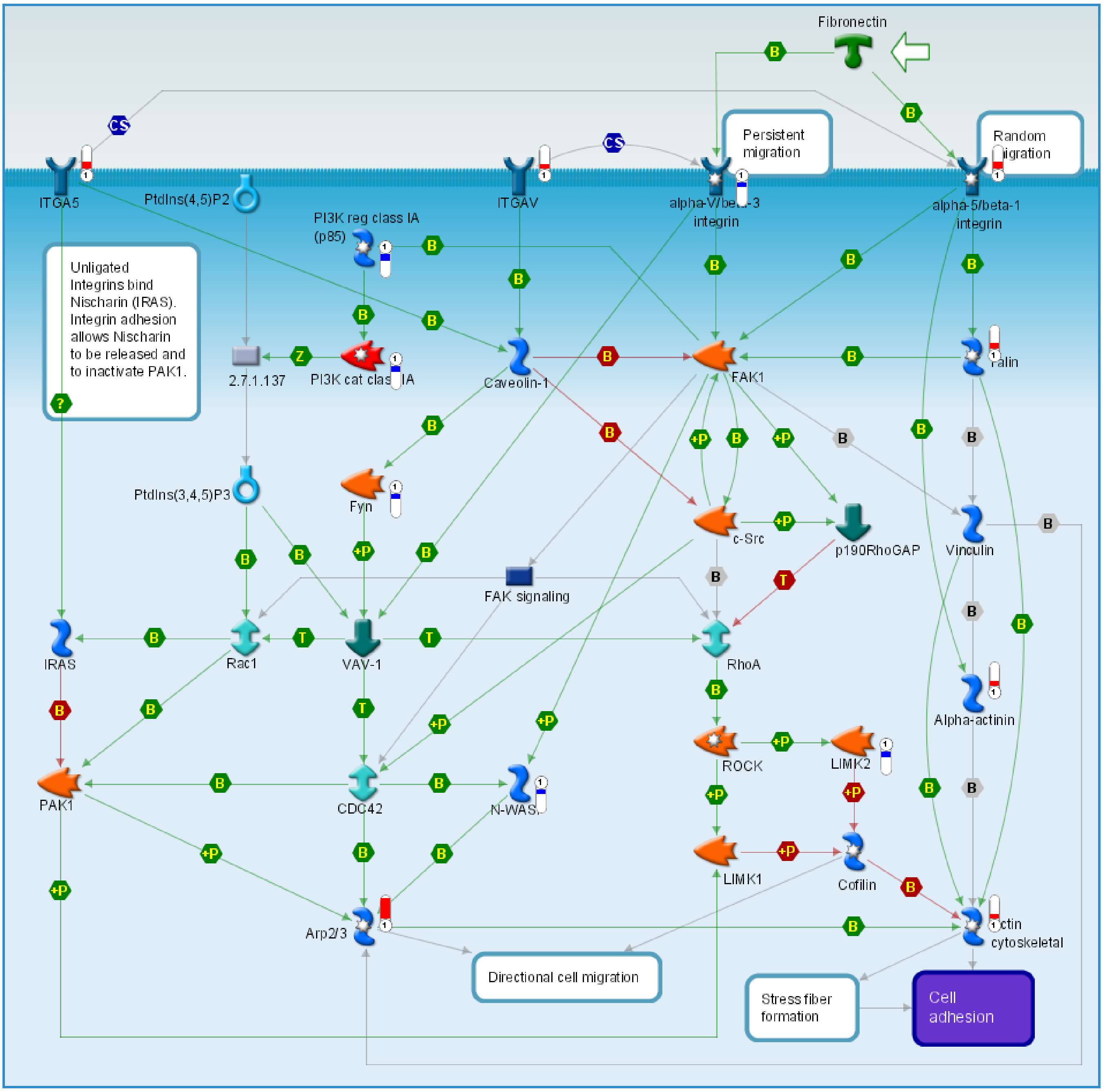

Gene Symbol	Protein	Class	Protein name	Fold Change
ACTB	ACTB_HUMAN		Actin, cytoplasmic 1	1.83
ACTG1	ACTG_HUMAN		Actin, cytoplasmic 2	
ACTN1	ACTN1_HUMAN		Alpha-actinin-1	
ACTN2	ACTN2_HUMAN		Alpha-actinin-2	

ACTN3	ACTN3_HUMAN		Alpha-actinin-3	
ACTN4	ACTN4_HUMAN		Alpha-actinin-4	1.97
ACTR2	ARP2_HUMAN		Actin-related protein 2	
ACTR3	ARP3_HUMAN		Actin-related protein 3	
ACTR3B	ACTR3B		ARP3 actin-related protein 3 homolog B (yeast)	
ARPC1A	ARC1A_HUMAN		Actin-related protein 2/3 complex subunit 1A	
ARPC1B	ARC1B_HUMAN		Actin-related protein 2/3 complex subunit 1B	
ARPC2	ARPC2_HUMAN		Actin-related protein 2/3 complex subunit 2	
ARPC3	ARPC3_HUMAN		Actin-related protein 2/3 complex subunit 3	
ARPC4	ARPC4_HUMAN		Actin-related protein 2/3 complex subunit 4	
ARPC5	ARPC5_HUMAN		Actin-related protein 2/3 complex subunit 5	9.43
CAV1	CAV1_HUMAN		Caveolin-1.	
CDC42	CDC42_HUMAN		Cell division control protein 42 homolog precursor	
CFL1	COF1_HUMAN		Cofilin-1	
CFL2	COF2_HUMAN		Cofilin-2	
FN1	FINC_HUMAN		Fibronectin precursor	
FYN	FYN_HUMAN		Proto-oncogene tyrosine-protein kinase Fyn	-1.93
GRLF1	GRLF1_HUMAN		Glucocorticoid receptor DNA-binding factor 1	
ITGA5	ITA5_HUMAN		Integrin alpha-5 precursor	2.82
ITGAV	ITAV_HUMAN		Integrin alpha-V precursor	1.93
ITGB1	ITB1_HUMAN		Integrin beta-1 precursor	
ITGB3	ITB3_HUMAN		Integrin beta-3 precursor	-2
LIMK1	LIMK1_HUMAN		LIM domain kinase 1	
LIMK2	LIMK2_HUMAN		LIM domain kinase 2	-2.5

LOC643751				
NISCH	NISCH		Nischarin	
PAK1	PAK1_HUMAN		Serine/threonine-protein kinase PAK 1	
PIK3CA	PK3CA_HUMAN		Phosphatidylinositol-4,5-bisphosphate 3-kinase catalytic subunit alpha isoform	-2.4
PIK3CB	PK3CB_HUMAN		Phosphatidylinositol-4,5-bisphosphate 3-kinase catalytic subunit beta isoform	2.01
PIK3CD	PK3CD_HUMAN		Phosphatidylinositol-4,5-bisphosphate 3-kinase catalytic subunit delta isoform	
PIK3R1	P85A_HUMAN		Phosphatidylinositol 3-kinase regulatory subunit alpha	-3.02
PIK3R2	P85B_HUMAN		Phosphatidylinositol 3-kinase regulatory subunit beta	-1.91
PTK2	FAK1_HUMAN		Focal adhesion kinase 1	
RAC1	RAC1_HUMAN		Ras-related C3 botulinum toxin substrate 1 precursor	
RHOA	RHOA_HUMAN		Transforming protein RhoA precursor	
ROCK1	ROCK1_HUMAN		Rho-associated protein kinase 1	
ROCK2	ROCK2_HUMAN		Rho-associated protein kinase 2	
SRC	SRC_HUMAN		Proto-oncogene tyrosine-protein kinase Src	
TLN1	TLN1_HUMAN		Talin-1.	2.31
TLN2	TLN2_HUMAN		Talin-2.	
VAV1	VAV_HUMAN		Proto-oncogene vav.	
VCL	VINC_HUMAN		Vinculin	
WASL	WASL_HUMAN		Neural Wiskott-Aldrich syndrome protein	-1.72
